# A chimeric protein-based vaccine elicits a strong IgG antibody response and confers partial protection against Shiga toxin-producing *Escherichia coli* in mice

**DOI:** 10.3389/fimmu.2023.1186368

**Published:** 2023-07-27

**Authors:** David A. Montero, Richard Garcia-Betancourt, Roberto M. Vidal, Juliana Velasco, Pablo A. Palacios, Daniela Schneider, Carolina Vega, Leonardo Gómez, Hernán Montecinos, Rodrigo Soto-Shara, Ángel Oñate, Leandro J. Carreño

**Affiliations:** ^1^ Programa de Inmunología, Instituto de Ciencias Biomédicas, Facultad de Medicina, Universidad de Chile, Santiago, Chile; ^2^ Instituto Milenio de Inmunología e Inmunoterapia, Facultad de Medicina, Universidad de Chile, Santiago, Chile; ^3^ Departamento de Microbiología, Facultad de Ciencias Biológicas, Universidad de Concepción, Concepción, Chile; ^4^ Programa de Microbiología y Micología, Instituto de Ciencias Biomédicas, Facultad de Medicina, Universidad de Chile, Santiago, Chile; ^5^ Unidad de Paciente Crítico, Clínica Hospital del Profesor, Santiago, Chile; ^6^ Programa de Formación de Especialista en Medicina de Urgencia, Universidad Andrés Bello, Santiago, Chile; ^7^ Plataforma Experimental, Facultad de Odontología, Universidad de Chile, Santiago, Chile; ^8^ Departamento de Biología Celular, Facultad de Ciencias Biológicas, Universidad de Concepción, Concepción, Chile

**Keywords:** Shiga toxin-producing *Escherichia coli*, STEC, O157:H7, chimeric protein, vaccine, diarrhea

## Abstract

**Background:**

Shiga toxin-producing *Escherichia coli* (STEC) is a foodborne pathogen that causes gastrointestinal infections, ranging from acute diarrhea and dysentery to life-threatening diseases such as Hemolytic Uremic Syndrome. Currently, a vaccine to prevent STEC infection is an unmet medical need.

**Results:**

We developed a chimeric protein-based vaccine targeting seven virulence factors of STEC, including the Stx2B subunit, Tir, Intimin, EspA, Cah, OmpT, and AggA proteins. Immunization of mice with this vaccine candidate elicited significant humoral and cellular immune responses against STEC. High levels of specific IgG antibodies were found in the serum and feces of immunized mice. However, specific IgA antibodies were not detected in either serum or feces. Furthermore, a significantly higher percentage of antigen-specific CD4+ T cells producing IFN-γ, IL-4, and IL-17 was observed in the spleens of immunized mice. Notably, the immunized mice showed decreased shedding of STEC O157:H7 and STEC O91:H21 strains and were protected against weight loss during experimental infection. Additionally, infection with the STEC O91:H21 strain resulted in kidney damage in control unimmunized mice; however, the extent of damage was slightly lower in immunized mice. Our findings suggest that IgG antibodies induced by this vaccine candidate may have a role in inhibiting bacterial adhesion and complement-mediated killing.

**Conclusion:**

This study provides evidence that IgG responses are involved in the host defense against STEC. However, our results do not rule out that other classes of antibodies also participate in the protection against this pathogen. Additional work is needed to improve the protection conferred by our vaccine candidate and to elucidate the relevant immune responses that lead to complete protection against this pathogen.

## Introduction

Shiga toxin-producing *Escherichia coli* (STEC) are a group of foodborne pathogens that cause diarrhea and dysentery. In some cases, these infections can lead to more severe and potentially deadly complications, such as Hemolytic Uremic Syndrome (HUS), mainly in children under 5 years old and the elderly (1).

STEC is commonly found in the intestines of ruminant animals, such as cattle and sheep, and can contaminate food products derived from these animals (2). Through the fecal excretion of these animals, STEC can contaminate other food products such as vegetables, fruits, and water sources. As a result, STEC outbreaks are associated with contaminated food, affecting public health and the food industry (3).

Globally, O157:H7 is the most common serotype of STEC associated with cases of HUS. The main virulence factor of STEC is the Shiga toxin (Stx). During infection, this toxin can spread systemically and cause damage to the kidneys and central nervous system, leading to HUS (4).

Adhesion and colonization of the intestine are key steps in STEC infection. A group of STEC strains, formerly called enterohemorrhagic *E. coli* (EHEC), harbor the pathogenicity island (PAI) called Locus of Enterocyte Effacement (LEE) (5). These strains are currently called LEE-positive STEC to differentiate them from other STEC strains that lack LEE (LEE-negative) (6). One of the genes located in the LEE PAI is the *eae* gene, which encodes Intimin, a non-fimbrial adhesin considered the main factor mediating the adherence of these bacteria (7). Additionally, the LEE PAI encodes the type three secretion system (T3SS), which facilitates the translocation of multiple proteins into the enterocytes. These proteins include the translocated intimin receptor (Tir) and other virulence factors that induce architectural and physiological changes in the intestinal epithelial cells through signal transduction mechanisms (1, 8). Adhesion mediated by the LEE PAI results in the attaching and effacement (A/E) lesion, characterized by a rearrangement of the cytoskeleton and the production of a pedestal-like structure at the site of bacterial attachment. This leads to the loss of intestinal microvilli, inflammation, and diarrhea (9–11). The LEE PAI is also carried by enteropathogenic *E. coli* (EPEC) and the murine pathogen *Citrobacter rodentium*, thus these bacteria known as “A/E pathogens.”

Studying the pathogenesis of STEC infection in human volunteers is not possible due to the risk developing HUS and dying. Ruminants do not develop diseases due to colonization by this bacterium. Although laboratory animals such as rabbits and mice have been used as models of STEC infection, none of them fully reproduces the symptoms and diseases observed in humans (12, 13). However, the streptomycin-treated mouse model is widely used for studying the virulence and colonization of different STEC strains. In this model, streptomycin is administered to mice through water before and during the infection, which reduces the intestinal microbiota and facilitates STEC colonization by decreasing competitive exclusion. Depending on the STEC strain, the dose and the route of inoculation, streptomycin-treated mice may exhibit renal damage and, in some cases, mortality (14).

Currently, there is no licensed vaccine against STEC for humans, and vaccines licensed for use in cattle reduce, but do not eliminate, the colonization and spread of these bacteria (15, 16). Furthermore, there is a growing interest in developing effective vaccines against STEC, especially considering the significant burden of illness and economic cost associated with these infections (16).

Several promising vaccine candidates for STEC have been developed based on proteins encoded in the LEE PAI (17). Examples include Tir, Intimin and structural proteins of the T3SS, such as EspA, EspB, EspD, and EscC (18–26). In addition to these conventional antigens, there is growing interest in exploring alternative vaccine approaches that target non-LEE-encoded antigens. For instance, the subunit B of the Shiga toxin 2 (Stx2B), and outer membrane proteins such as LomW, OmpT, Cah and Hes have gained attention as potential antigens for vaccine development (20, 22, 25–29).

The biotechnological revolution, particularly the advancements in gene synthesis, has opened new doors in the rational design of more efficient and cost-effective vaccines (30–34). Vaccines that contain multiple antigens are thought to offer more comprehensive protection than those with fewer antigens. The rationale behind this is that vaccines with a wider range of antigens can stimulate a more varied and diverse immune response, resulting in a more effective defense against different strains or newly emerging strains of the target pathogen (35–39). One promising strategy is the use of chimeric proteins that carry selected epitopes from various strains or pathogens. This approach has shown the potential to enhance the immunogenicity of the recombinant antigen and the possibility of eliciting a broader immune response (28, 32, 39).

In this study, we developed two chimeric proteins containing antigenic domains from seven virulence factors of STEC, including Stx2B, Tir, Intimin, AggA, EspA, Cah, and OmpT proteins. These chimeric proteins were combined and formulated with the AddaVax™ adjuvant (Invivogen, USA). Subsequently, we evaluated the potential of these chimeras as vaccine candidates against STEC using the streptomycin-treated mouse model. Overall, our results show that this vaccine formulation elicited high levels of specific IgG antibodies and provided partial protection against STEC, including the LEE-positive O157:H7 and the LEE-negative O91:H21 strains. These results highlight the potential of chimeric proteins for the development of new vaccines against bacterial pathogens.

## Materials and methods

### Bacterial strains and growth conditions

In this study, we used spontaneously derived streptomycin-resistant (Str^r^) mutants of STEC O157:H7 86-24 and STEC O91:H21 V07-4-4 strains, which were obtained from previous studies (28, 40). Bacterial cultures were routinely grown at 37°C in either Luria-Bertani (LB) broth (Cat #244620, BD Difco™) or Low-glucose Dulbecco’s modified Eagle medium (DMEM, HyClone™), supplemented with streptomycin as necessary.

### Design of chimeric proteins

The design and *in silico* modeling of chimeric proteins involved the prediction of B-cell epitopes using several tools available at the Immune Epitope Database (IEDB) server (41). These tools included BepiPred 2.0 (42) with a threshold value set at 0.5, and the Kolaskar and Tongaonker antigenicity method (43) with a threshold value of 1.0 and a window size of 7. Additionally, prediction of peptides binding to MHC-II molecules was performed using the NetMHCIIpan 4.1 method (44). For this, a set of seven human HLA-DR alleles were analyzed: HLA-DRB1*03:01, HLA-DRB1*07:01, HLA-DRB1*15:01, HLA-DRB3*01:01, HLA-DRB3*02:02, HLA-DRB4*01:01, HLA-DRB5*01:01. The default parameter of an epitope length of 15 amino acids was defined.

The predicted three-dimensional structure of Chimera 3 (Chi3) and Chimera 4 (Chi4) was constructed using RaptorX (45) and Phyre2 (using intensive modelling mode) (46). The quality evaluation and validation of the models were performed using RAMPAGE (47) and PROSA-web (48). The chemical and physical properties of the chimeric proteins were predicted using Protein-Sol (49) and ProtParam (50). The modeled structures were visualized with UCSF Chimera X (51).

### Purification and antigenicity of chimeric proteins

Synthetic genes coding for Chi3 and Chi4 proteins were ordered from GenScript Biotech (USA). The nucleotide sequences were optimized for *E. coli* expression and cloned into the pET52b(+) vector (Cat #71554, Novagen^®^, Merck) with an N-terminal 10xHis-tag. The ClearColi^®^ BL21(DE3) strain (LGC, Biosearch Technologies, Hoddesdon, UK) was then transformed with recombinant plasmids containing synthetic genes. To purify recombinant proteins, transformant *E. coli* strains were grown in LB-Miller Medium containing ampicillin (100 µg/ml) at 37°C. When the culture reached an optical density ~1.2 at 600 nm, it was supplemented with 1 mM IPTG for 4 h. Subsequently, bacteria were harvested by centrifugation and resuspended with lysis buffer (B-PER™, Cat #89821, Thermo Scientific, USA) followed by sonication. The pellet obtained after centrifugation was dissolved using 8M urea. Denatured chimeric proteins were further purified by using a Ni-column (GE17- Cat #5248-01, HisTrap™ High Performance, Merck) in an AKTA FPLC system (GE Healthcare, USA). The purified chimeric proteins were refolded and sterilized using a 0.22 μm filter before being stored in aliquots at -20°C until use. The concentration was determined by Bradford protein assay (Cat #23236, Thermo Scientific, USA) with BSA as standard. Standard SDS-PAGE and Western blot confirmation determined the protein purity and molecular weight. In addition, the reactivity of Chimeric proteins to IgG and IgA present in human and cattle sera was assessed by ELISA assay, as previously described (28, 29). Human and cattle sera were obtained from previous works (29, 52), with approved certificates of ethics and bioethics.

### Formulation of the chimeric protein-based vaccine candidate

In general, vaccines containing multiple antigens have shown to enhance protective efficacy compared to those containing fewer antigens (28, 36, 37). Hence, we combined Chi3 and Chi4 proteins into a single formulation, anticipating that this approach would enhance the efficacy and coverage of the vaccine against different serotypes of STEC. Thus, a mixture of 25 µg of each chimera was adsorbed onto the adjuvant AddaVax™ (Cat. code vac-adx-10, Invivogen, USA), resulting in the vaccine formulation that was used in subsequent studies. AddaVax™ is an oil-in-water adjuvant, based on the MF59 formula [i.e., squalene oil (5% v/v or 4.29% w/v based on squalene density of 0.858 (PubChem CID: 638072)], Tween 80 (0.5% w/v) and Span 85 (0.5% w/v) in citrate buffer (10 mM, pH 6.5)), which has been licensed in Europe for use in flu human vaccines. In addition, AddaVax™ promotes a more balanced Th1/Th2 response than that obtained with alum (53–55).

### Ethics statement

All animal experiments were performed at Plataforma Experimental, Facultad de Odontología, Universidad de Chile, Santiago, Chile, following protocols and bioethical guidelines approved by the Comité Institucional de Cuidado y Uso de Animales (CICUA) (Protocol 19250-ODO-UCH). Female BALB/c mice (5−6 weeks old) were housed in a pathogen-free and biosafety level 2 facility with water and food *ad libitum* and maintained on a 12-h light cycle. Mice were monitored on a daily basis using a score system to assess the perception of pain, distress, and discomfort. The scoring sheet employed was a modified version of the supervision protocol proposed by Morton and Griffiths, 1985 (56). Briefly, five parameters were evaluated: weight loss, appearance, spontaneous behavior, behavior in response to handling, and pathologies resulting from STEC infection. Specifically, scores from 0 to 3 were assigned, indicating normal to severe clinical signs, respectively. Mice were euthanized if their scores ranged from 10 to 15 (**Supplementary Table 1**).

### Immunization studies

**Figure 1:**
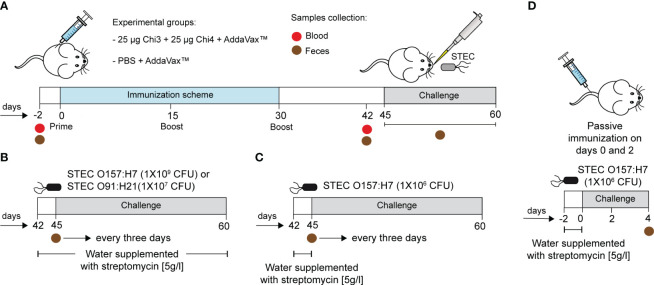
Immunization scheme and infection experiments. **(A)** BALB/c mice were immunized intramuscularly with a formulation containing 25 µg of Chi3 and 25 µg of Chi4 plus AddaVax™ adjuvant. Two booster doses of the same formulation were given every 15 days. The control group was given PBS plus AddaVax™ adjuvant. Blood and fecal samples were collected 2 days before the experiment started and 12 days after the last booster (day 42). **(B, C)** Two different infection protocols were used. Briefly, two days prior to infection, mice were treated with streptomycin to reduce the intestinal microbiota and promote colonization by streptomycin-resistant challenged strains. In the first infection protocol **(B)**, streptomycin administration was maintained throughout the experiment. In the second infection protocol **(C)**, streptomycin administration was performed only two days prior to infection. The infective doses are indicated. Fecal samples were collected every 3 days to assess the shedding of the inoculated bacteria. **(D)** Passive immunization experiments and challenge. Mice were passively immunized *via* the tail vein with 200 µg of anti-Chi3/Chi4 IgG, and two days later, with a similar dose. The control group received similar doses but of IgG from control mice (PBS + AddaVax™). Four days after infection, mice were euthanized, and the cecum was collected to determine the level of colonization of the challenged strain.

Two types of immunization studies were performed, one active and one passive (**Figure 1**). For active immunization experiments, fifty female BALB/c mice (5−6 weeks old) were randomly distributed into two experimental groups. One group of mice (n=25) was immunized by the intramuscular (i.m.) route with 50 µl of a vaccine formulation containing 25 µg of each chimeric protein (i.e., Chi3 and Chi4) plus 25 µl of the adjuvant AddaVax™. The other group of mice (n=25) received PBS plus adjuvant and served as the control group. Two booster doses were administered with the same formulations on days 15 and 30 (**Figure 1A**). To measure the humoral response, two days before the initial immunization and two weeks after the last boost, five mice per group were used to collect 0.2 - 0.3 ml of blood by submandibular bleeding method (57). Stool samples were also collected during these time points. Blood samples were allowed to clot at 37°C for 30 minutes and then centrifuged at 1000 x g for 10 minutes. The supernatant (serum) was collected, filtered through a 0.22 µm cellulose acetate syringe filter, aliquoted and stored at -80°C until utilized in the adherence inhibition assay, serum bactericidal assay, and passive immunization experiments. Fecal samples were weighed and homogenized (100 mg/ml) in PBS containing 25 mM EDTA, 0.1 mg/ml soybean trypsin inhibitor (Cat #17075029, ThermoFisher Scientific), and 2 mM PMSF (Cat #36978, ThermoFisher Scientific). The fecal suspension was subjected to an initial centrifugation at 15,000 x g for 5 min at a temperature of 4°C. Subsequently, the resulting supernatant was carefully collected and subjected to a second round of centrifugation under the same conditions, but for up to 15 min. Subsequently, this final supernatant was stored at -80°C until use. For measurement of cellular response, two weeks after the final boost, five mice per group were euthanized, terminally bleed, and their spleens collected under aseptic conditions. The remaining twenty mice per group were used in challenge studies (**Figures 1B, C**).

For passive immunization experiments, a pool of sera was obtained from five actively immunized mice (Chi3 + Chi4 + AddaVax™) two weeks after the last booster immunization. As a control, another set of sera was obtained from five control mice (PBS + AddaVax™). IgG was then purified with the Melon™ Gel IgG Spin Purification kit (Cat #45206, ThermoFisher Scientific) and the concentration of purified IgG was determined using the ELISA Flex kit – Mouse IgG (Cat #3825-1AD-6, Mabtech, USA). Then, ten female BALB/c mice (10−11 weeks old) were randomly distributed into two experimental groups (n=5 for each group). One group of mice was passively immunized, through the tail vein, with 200 µg of anti-Chi3/Chi4 IgG and two days later with a similar dose. The other group of mice served as control and received 200 µg of control IgG (**Figure 1D**).

### Measurement of humoral response

Flat-Bottom 96-well ELISA plates (Cat #442404, ThermoFisher Scientific) were incubated with a mixture of 1 µg of each chimeric protein diluted in 100 µl of Phosphate-Buffered Saline (PBS; pH 7.2) overnight at 4°C. Then, they were incubated with 300 µl/well of blocking solution (T-PBS + 1% bovine serum albumin) for 1 h at 37°C. The plates were washed 3 times with 300 µl/well of PBS containing 0.05% Tween 20 (T-PBS). Mice sera or fecal supernatants were serially diluted in blocking solution (100 µl/well) and incubated for 1h at 37°C. After 4 washes with T-PBS (400 µl/well), secondary antibody-HRP conjugate anti-mouse IgG (Cat #610-1302), anti-mouse IgG1(Cat #610-103-040), anti-mouse IgG2a (Cat #610-103-041), anti-mouse IgG2b (Cat #610-103-042), anti-mouse IgG3 (Cat #610-103-042), or anti-mouse IgA (Cat #610-4306), all from Rockland Immunochemicals (USA), were diluted 1:2000 in blocking solution (100 µl/well) and incubated for 60 min at room temperature (RT). After 4 washes with Tris-buffered saline (TBS) (400 µl/well; pH 7.5) containing 0.05% Tween 20, TMB (3,3’,5,5’-Tetramethylbenzidine) substrate was incubated for 10 min at RT. The reaction was stopped with 2N H_2_SO_4_ (50 µl/well). The absorbance of the solution in each well was determined at 450 nm using a Synergy HT microplate reader (Biotek Instruments, USA). The results were reported as the reciprocal of the highest titer, giving an optical density (OD) reading of at least the mean ±2 standard deviations compared to the baseline sera or fecal supernatants. Each sample was determined twice in duplicate, and results were shown as the mean reciprocal endpoint titer.

### Measurement of cellular response

Spleens from immunized or control mice were collected two weeks after the last immunization. Then, cell suspensions were obtained by using a 40 µm cell strainer (Cat#CLS431750, Sigma-Aldrich, USA) and cold RPMI (Gibco, USA) supplemented with 10% FBS and 1% penicillin-streptomycin), after removing erythrocytes by Red Blood Cell (RBC) Lysis Buffer (Cat #420302, Biolegend, USA). Splenocytes were cultured in V-Shaped-Bottom 96-well plates (Cat #EW-01728-80, Costar) (approximately 4 X 105 cells/well) at 37°C, 5% CO2 in RPMI supplemented with 10% FBS and 1% penicillin-streptomycin. Then, splenocytes were stimulated *in vitro* with heat-killed STEC O157:H7 (2 μg/well). After 72 hours, cells were collected and washed with FACS stain buffer (1X PBS, 2% Fetal Bovine Serum and 0.05% sodium azide). Cells were incubated with purified rat anti-mouse CD16/32 antibody (clone 2.4G2, Cat #553142, BD Biosciences, USA) to block non-specific binding to FcɣR. Subsequently, the staining was carried out with the following specific anti-mouse monoclonal antibodies: TCR β-FITC (Clone H57-597, Cat #109206), IL4-PE Cyanine 7 (Clone 11B11, Cat # 504118), CD4-APC Cyanine 7 (Clone GK1.5, Cat #100414) (all from Biolegend, USA); IFNg-Alexa Fluor 700 (Clone XMG 1.2, Cat #557998), IL17A-PE, CF594 (Clone TC11-18H10, Cat #562542), (all from BD Biosciences, USA). For intracellular antigen staining, the cells were permeabilized with Foxp3/Transcription Factor Staining Buffer (Cat #50-112-8857, Invitrogen, USA). For detection of cytokines expressed in CD4+ T cells, *ex vivo* stimulation of splenocytes was performed with phorbol 12-myristate 13-acetate (PMA, 50 ng/mL) (Cat #P1585-1MG, Sigma-Aldrich, USA) and Ionomycin (4 µg/mL) (Cat # 56092-82-1, Sigma-Aldrich, USA) for four hours at 37°C in the presence of Monensin (4 µg/mL) (Cat # 00-4505-51, Thermo Fisher Scientific, USA), and Brefeldine A (4 µg/mL) (Cat #00-4506-51, Thermo Fisher Scientific, USA). To analyze the percentage of cytokines expressed in CD4+ T cells, we adjusted the gate according to the fluorescence minus one (FMO) control. The flow cytometry assay was performed in an 18-color flow cytometer (BD LSR FORTESSA X-20). The analysis from acquired cells was carried out in the FlowJo V10 software (FlowJo LLC, USA)

### Outer membrane vesicles purification, transmission electron microscopy and immunogold labeling

The OMVs were isolated as previously described (58), with modifications. Briefly, the STEC O157:H7 86-24 strain was cultured in 500 ml of LB broth for 15 h at 37°C with shaking (180 rpm). The culture was centrifuged at 10,000 x g for 10 min at 4°C, and the supernatant was passed through a 0.22 µm filter. The filtered supernatant was concentrated using Amicon^®^ Ultra 15 ml tubes (3,000 Da cutoff, Cat #UFC9003, Merck Millipore, USA) and subsequently ultracentrifuged at 235,000 x g for 2 h at 4°C. The pellet (OMVs) was resuspended in 1 ml of 20 mM TRIS-HCl (pH 8.0) and stored at -80°C until use.

TEM visualization and immunogold labeling were performed as previously described (59), with modifications. Briefly, 10 µl of an overnight culture of STEC O157:H7 86-24 or purified OMVs (dissolved in PBS) were absorbed onto copper grids for 5 minutes at 37°C. After three washes with PBS, the samples were fixed with 2.0% glutaraldehyde and negatively stained with 0.5% phosphotungstic acid. Next, the grids were incubated with 1% BSA-0.01 M glycine in PBS for 1 hour at room temperature. Following this, the grids were incubated with a pool of sera obtained from five immunized mice or five control mice, diluted 1:100 in a solution of 0.2% BSA-0.05% Tween 20 (T-BSA), for 1 hour at room temperature. After three washes with T-BSA, the grids were incubated with 10 nm gold particle-conjugated mouse anti-IgG (Cat #G7652, Sigma-Aldrich, USA) at a dilution of 1:100. Finally, the samples were washed three times and analyzed using a Philips Tecnai 12 microscope at the Electron Microscopy Laboratory, Faculty of Biological Sciences, Pontificia Universidad Católica de Chile, Chile.

### Challenge studies

Two different infection protocols were carried out using the streptomycin-treated mouse model (60).

In the first infection protocol (**Figure 1B**), two weeks after the last booster immunization (day 45), five mice per group received *ad libitum* water containing streptomycin (5 g/l) for two days prior to infection. In addition, the stool was confirmed to be free of streptomycin-resistant *E. coli* at the time of infection. Bacterial strains were grown overnight in low-glucose DMEM medium containing 50 µg/ml streptomycin at 37°C with shaking. Before inoculation, food and water were restricted overnight (12 h). The next morning, mice were inoculated orally with 50 µl of bacterial suspension containing 1×10^9^ CFU of STEC O157:H21 86-24 strain or 1×10^7^ CFU of STEC O91:H21 V07-4-4 strain, which are non-lethal doses, according to previous studies (40) .After challenge, food and water supplemented with streptomycin were reintroduced *ad libitum* throughout the experiment. In the second infection protocol (**Figure 1C**), streptomycin administration was performed only two days prior to infection, and the infective dose was 1×10^6^ CFU of STEC O157:H21 86-24 strain.

Fecal shedding of the challenged strains was recorded every three days. For this, 100 mg of feces were suspended and homogenized in 1 ml of PBS. The suspension was then centrifuged at 50 x g for 1 min to precipitate particles, and serial dilutions of the supernatant were plated on MacConkey or CHROMagar™ O157 agar plates supplemented with streptomycin (50 µg/ml). The weight of the mice was recorded every three days.

Within the second challenge study, the passively immunized mice were inoculated orally following the second infection protocol (**Figure 1D**). To determine the intestinal colonization of the challenge strain, these mice were euthanized four days after infection, and the cecum was collected under aseptic conditions. The cecal content was suspended in PBS, homogenized, and serially diluted. The dilutions were plated on MacConkey or CHROMagar™ O157 agar plates supplemented with streptomycin (50 µg/ml).

### Histopathological analysis of kidney tissue

Mice inoculated with the STEC O91:H21 V07-4-4 strain were euthanized 15 days post-infection. The kidneys were collected, fixed in 10% formaldehyde (pH 6.9), embedded in paraffin wax, and sectioned at 5 μm. The sections were then stained with hematoxylin and eosin (H/E). Pathological evaluation of the H/E-stained tissue sections was carried out by a pathologist blinded to the experimental design.

### Adherence inhibition assay

Caco-2 cells were cultured in 24-well plates (approximately 4 X 10^5^ cells/well) at 37°C, 5% CO_2_ in DMEM supplemented with 10% FBS and 1% penicillin-streptomycin. The bacterial strains (STEC O157:H7 or STEC O91:H21) were grown overnight in low glucose DMEM at 37°C with agitation. The next day, an aliquot was diluted 1:50 in the same medium and incubated at 37°C for 3-4 h with agitation. The serum from five mice immunized with Chi3 + Chi4 + AddaVax™ was pooled and inactivated at 56°C for 45 min. Sera from five non-immunized mice (PBS + AddaVax™) were pooled, inactivated, and used as a control. Next, an inoculum of ~10^6^ CFU of the bacteria was incubated in DMEM medium or DMEM medium containing inactivated hyperimmune or control serum at a 1:5 dilution for 30 min at 37°C with gentle agitation. After that, the bacteria were used to infect Caco-2 cells for 2 h at 37°C with 5% CO_2_. Five washes with PBS removed non-adherent bacteria, and adherent bacteria were recovered by lysis with 0.1% Triton X-100, serially diluted in PBS, and plated on MacConkey agar plates for enumeration. The result was expressed as the percentage of adherent bacteria in relation to the number of bacteria added, normalized with the percentage of adherent bacteria that were incubated in DMEM medium (without serum). The assay was performed twice in duplicate.

### Serum bactericidal assay

Serum bactericidal assays were performed as described (26), with modifications. An inoculum of approximately 10^6^ CFU of bacteria (STEC O157:H7 or STEC O91:H21) was incubated with sera from immunized mice or control sera at a dilution of 1:5, either active or inactivated, and inactivated sera supplemented with exogenous mouse complement (Cat #S3269-5ML, Sigma-Aldrich, USA). The incubation was for 1 h at 37°C with slight agitation. Surviving bacteria were determined by serial dilutions and plating on MacConkey agar plates. The serum bactericidal activity was calculated using the equation: (bacterial CFU in control sera – bacterial CFU hyperimmune sera)/bacterial CFU in control sera X 100. The assay was performed twice in duplicate.

### Statistical analysis

All statistical analysis was done using GraphPad Prism software (v9.0) (GraphPad, Dotmatics, USA). For all statistical tests, a P value of <0.05 was considered significant. Statistical differences in humoral and cellular responses between groups were determined by Mann-Whitney test. Statistical differences in bacterial colonization and body weighs between groups were determined by two-way ANOVA test, followed by Sidak’s multiple comparison test. Statistical differences between groups in the histological analysis were determined by two-tailed Student’s t test. Statistical differences between groups in the adhesion assay experiments were determined by Mann-Whitney test. Statistical differences between groups in the Serum bactericidal activity assay were determined by Kruskal-Wallis test.

## Results

### Antigen selection and rationale design of chimeric proteins

For the rational design of a chimeric protein-based vaccine against STEC, antigenic proteins previously evaluated as immunogens in a mouse model were prioritized. Among these proteins are the B subunit of the Shiga toxin 2 (Stx2B), Tir, Intimin, AggA, EspA, Cah and OmpT. **Supplementary Table 2** indicates the biological function and immunogenic properties that have been previously reported for these proteins.

Subsequently, in order to predict linear B and T cell epitopes, these proteins were analyzed *in silico* using several immunobioinformatics tools, as described in material and methods section. As a result, several epitopes were predicted, and some of them were located consecutively or partially overlapping, suggesting the existence of antigenic domains (**Supplementary Tables 3**-**9**). Based on these results, two chimeric proteins containing several antigenic domains were designed and named Chimera 3 (Chi3) and Chimera 4 (Chi4), following the nomenclature used in a previous study in which two other chimeric proteins were developed (Chimera 1 and Chimera 2) (28).

Chi3 is a 479-aa protein with a theoretical molecular mass of 51.9 KDa that contains antigenic domains of the stx2B subunit and the Tir, AggA and Intimin proteins (**Supplementary Table 10**). Chi4 is a 39-aa protein with a theoretical weight of 41.5 KDa that contains antigenic domains of the Cah, EspA, and OmpT proteins (**Supplementary Table 11**). Additionally, through *in silico* tools, it was predicted that both chimeric proteins are soluble and stable *in vitro*, suggesting that their production and purification are feasible (**Table 1**).

**Table 1:** Antigenic domains and chemical and physical properties of the designed fusion proteins.

Protein	Antigenic domains (No. of amino acids)	MW (KDa)	Theoretical pI	Solubility^1^	Estimated half-life^2^	Stability index^3^
Chimera 3 (Chi3)	Stx2B (70) Tir (87) AggA (139) Intimin (183)	51.9	5.63	0.460	>10 h	30.98
Chimera 4 (Chi4)	Cah (190) EspA (66) OmpT (135)	41.5	5.04	0.546	>10 h	21.99

^1^ Prediction by Protein-Sol tool (49). Solubility scale (0–1). A value greater than 0.45 predicts that the protein is soluble. ^2^ Prediction of the time it takes for half of the amount of protein in *E. coli* to disappear after its synthesis. ^3^ The instability index provides an estimate of the stability of a protein in a test tube. A protein whose instability index is smaller than 40 is predicted as stable (61).

We predicted the 3D structures for Chi3 and Chi4 proteins using the RaptorX (45) and Phyre2 servers (46) (**Figures S1A, D**). Subsequently, analysis of the 3D models using RAMPAGE (47) indicated that the majority (>97%) of amino acid residues were located in favorable or allowed regions (**Figures S1B, E**). Additionally, analysis of the 3D models using PROSA-web (48) indicated that they have “Z” values within the optimal validation range (**Figures S1C, F**). These results suggest that the 3D models of Chi3 and Chi4 correspond to stable structures and have highly probable conformations.

### Production of chimeric proteins and determination of their antigenicity

**Figure 2:**
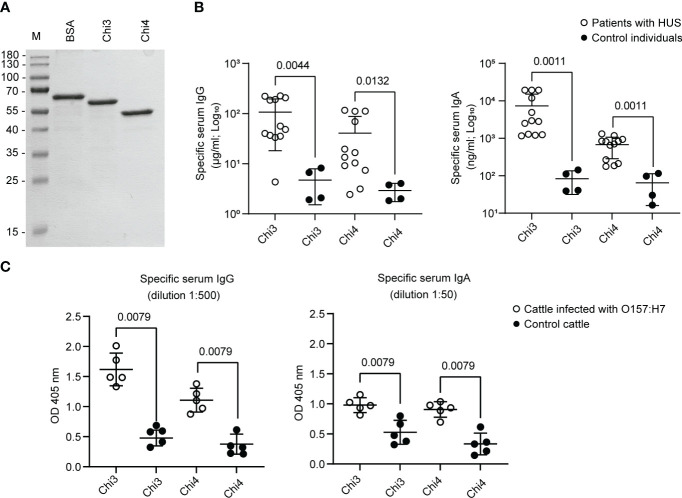
Synthesis of Chimera 3 and Chimera 4 proteins and determination of their antigenicity. **(A)** SDS-PAGE electrophoresis was performed to separate and analyze purified Chi3 and Chi4 proteins. Approximately 1 µg of protein was loaded in each lane. Bovine serum albumin (BSA) was used as a loading control. M indicates the molecular weight standard. **(B)** IgG and IgA antibodies reactive to Chi3 and Chi4 proteins were determined in the sera of patients infected with STEC O157:H7 (n=12) who developed Hemolytic Uremic Syndrome (HUS). Sera from uninfected individuals (n=4) were used as a control. Each point represents the concentration of immunoglobulin in serum determined by ELISA. The mean and standard deviation are shown. **(C)** IgG and IgA antibodies present in sera of cattle experimentally infected with STEC O157:H7 (n=5) and control cattle (n=5), which are reactive to Chi3 and Chi4 proteins. Each point represents the levels of specific immunoglobulin at the indicated dilution, determined by ELISA at an optical density (OD) of 405 nm. The mean and standard deviation are shown. Statistical differences and the *P* value obtained by the Mann-Whitney test are shown.

Consistent with the *in-silico* analyses, both proteins were produced in the ClearColi^®^ BL21(DE3) strain and subsequently purified by column chromatography. In addition, analysis of these proteins by SDS-PAGE indicated that they are stable and have the predicted molecular weight (**Figure 2A**).

Additionally, it was confirmed that Chi3 and Chi4 are reactive to IgG and IgA present in sera from patients infected with STEC O157:H7 (**Figure 2B**). On the contrary, the reactivity of these proteins to IgG and IgA present in sera of control (uninfected) individuals was basal and significantly lower. Similarly, it was determined that Chi3 and Chi4 proteins are reactive to IgG and IgA present in sera from cattle that were experimentally infected with STEC O157:H7. However, in control cattle (not infected), the reactivity of IgG and IgA to these proteins was significantly lower (**Figure 2C**). Together, these results demonstrated the feasibility of producing Chi3 and Chi4, as well as their antigenicity in humans and cattle that were infected with STEC O157:H7.

### Humoral immune response of immunized mice

Prior to immunizations, it was confirmed that mice did not have IgG and IgA antibodies reactive to Chi3 and Chi4 in their serum and feces (not shown). Subsequently, mice were immunized following the protocol described in **Figure 1A**. In the control group, mice were given PBS plus AddaVax™ following the same administration protocol.

**Figure 3:**
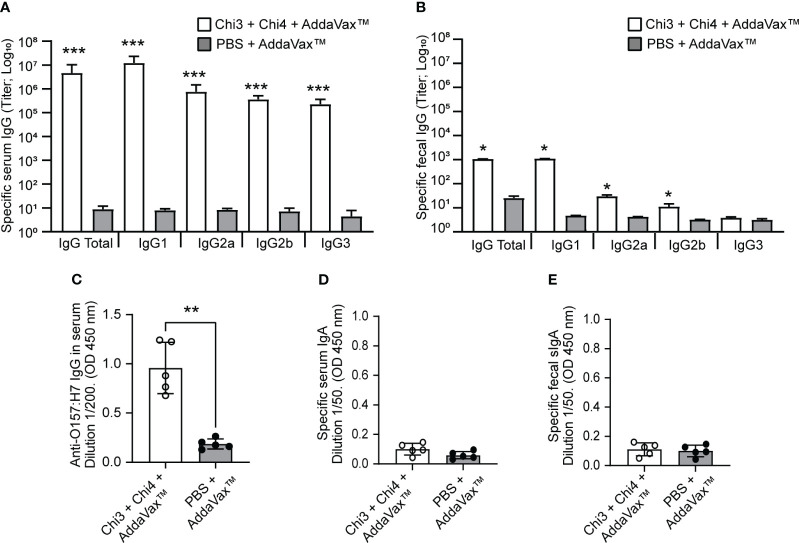
Humoral immune response generated by the immunization with chimeric proteins. Blood and fecal samples were obtained on day 42 from five mice per group to determine the humoral response by ELISA assay. **(A, B)** Anti-Chi3/Chi4 IgG antibody titers in serum and feces, respectively. The results are reported as the reciprocal of the highest titer, giving an optical density (OD) reading of at least the mean ±2 standard deviations compared to the baseline sera or fecal supernatants. **(C)** IgG in sera that is reactive to heat-killed O157:H7. Each point represents the levels of anti-O157:H7 immunoglobulin at the indicated dilution, determined by optical density (OD) at 450 nm. **(D)** and e) Anti-Chi3/Chi4 IgA antibodies in serum and feces, respectively. Each point represents the levels of specific immunoglobulin at the indicated dilution, determined by optical density (OD) at 450 nm. Error bars represent the standard deviation. Differences between groups were analyzed using the Mann-Whitney test and P values are indicated for significant differences (*P < 0.05, **P < 0.005, ***P < 0.0005).

Notably, after three doses of the vaccine formulation, mice generated significantly higher levels of IgG anti-Chi3/Chi4 antibodies in both serum and feces compared to control mice (**Figures 3A, B**). In serum, all IgG isotypes (IgG1, IgG2a, IgG2b, and IgG3) showed statistically significant titers of anti-Chi3/Chi4 antibodies. In feces, the IgG1 isotype showed the highest titer of anti-Chi3/Chi4 antibodies, followed by IgG2a and IgG2b, with all three being statistically significant compared to the control group. However, the IgG3 isotype was nearly undetectable, and no differences were found between the groups.

**Figure 4:**
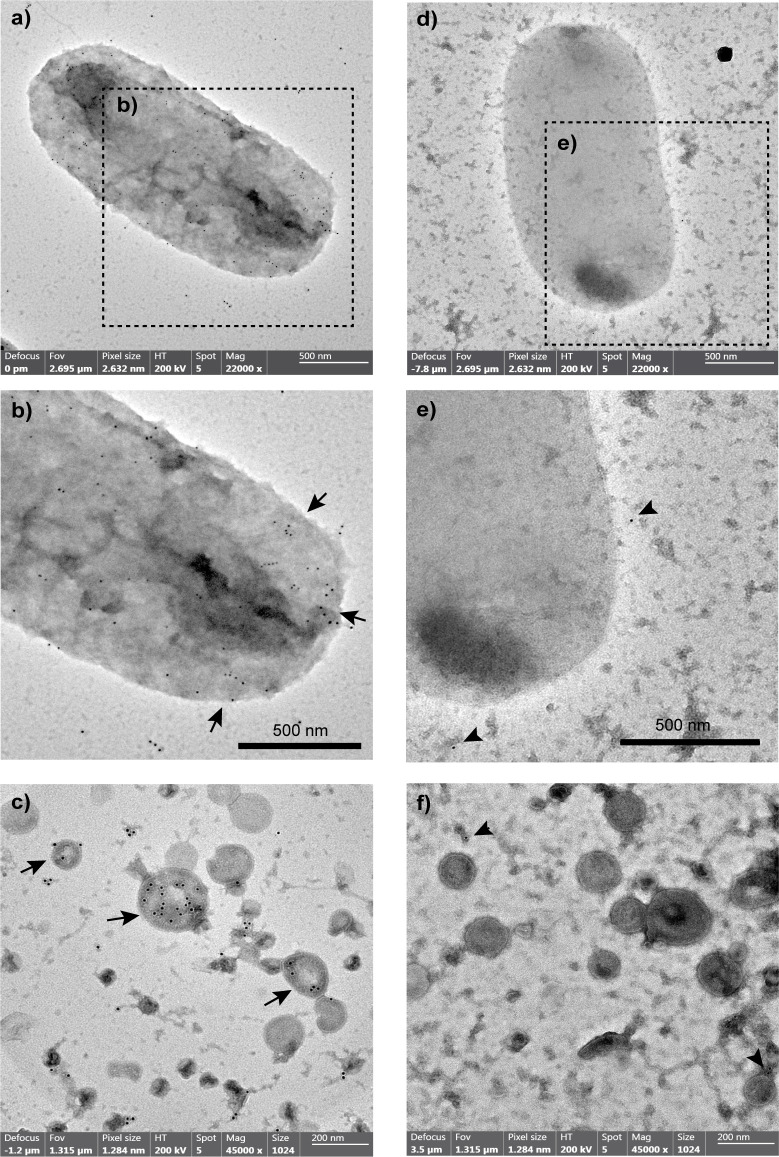
Reactivity of anti-Chi3/Chi4 IgG antibodies to antigens located on the surface of STEC O157:H7 and derived outer membrane vesicles (OMVs). The images correspond to transmission electron microscopy and immunolabeling with gold particles. For this, bacteria and OMVs were incubated with serum from mice immunized with Chi3 + Chi4 (dilution 1:100) or from control mice (dilution 1:100), followed by mouse anti-IgG conjugated with gold particles (10 nm in size, dilution 1:100). Antibodies produced by immunization with the vaccine formulation recognize antigens on the surface of O157:H7 **(A, B)** and OMVs **(C)**, with abundant labeling with gold particles (Arrows). In contrast, after incubation with control serum, only a few gold particles were observed (arrowheads) that appear to correspond to background noise rather than specific detection of antigens in the bacteria **(D, E)** or their OMVs **(F)**.

It was also found that immunization with the vaccine formulation generated IgG antibodies that were reactive to a bacterial lysate (heat-killed) of STEC O157:H7 (**Figure 3C**). The specificity of these antibodies against STEC O157:H7 was also demonstrated by transmission electron microscopy and immunogold labeling. In particular, the IgG anti-Chi3/Chi4 antibodies recognized antigens present on the surface of O157:H7 and outer membrane vesicles (OMVs) derived from this bacterium (**Figure 4**).

In marked contrast to the IgG antibody response, the levels of anti-Chi3/Chi4 IgA antibodies in serum and feces (secretory IgA; sIgA) were almost undetectable, and no differences were observed between groups (**Figures 3D, E**). Taken together, these results indicate that the vaccine formulation generates a strong humoral response characterized by the production of specific IgG antibodies.

### Cellular immune response of immunized mice

**Figure 5:**
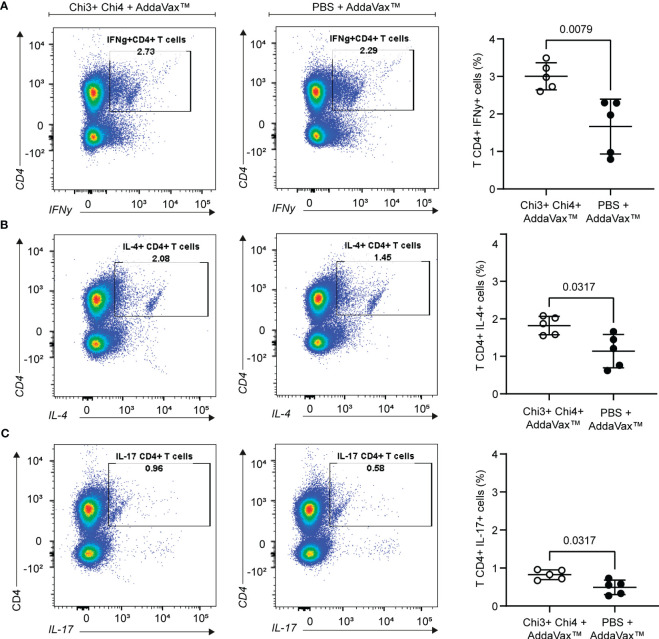
Cellular immune response generated by the immunization with chimeric proteins. Splenocytes were obtained from 5 mice per group on day 42 and then restimulated for 72 hours with 10 µg of heat-killed STEC O157:H7. Expansion of IFNγ+ **(A)**, IL-4+ **(B)**, and IL-17+ **(C)** CD4+ T cells was measured by intracellular staining. The left panels show a representative dot plot cytometry, while the graphs on the right show the means and standard deviation. Statistical differences and *P* value obtained by Mann-Whitney test are shown.

We next evaluated the cellular immune response generated by the vaccine formulation. For this, splenocytes from immunized and control mice were obtained, cultured *in vitro* and then restimulated with heat-killed STEC O157:H7 for 3 days. As shown in **Figure 5**, immunized mice exhibited robust responses of interferon gamma (IFNγ)-secreting CD4 T cells compared to the responses of mice that received only the adjuvant. Similarly, a significantly higher response of interleukin 4 (IL-4) and interleukin 17 (IL-17)-secreting CD4 T cells was observed in immunized mice compared to the control group. Together, these results highlight the ability of the vaccine formulation to generate specific cellular responses against STEC O157:H7.

### Evaluation of the protection against STEC O157:H7

**Figure 6:**
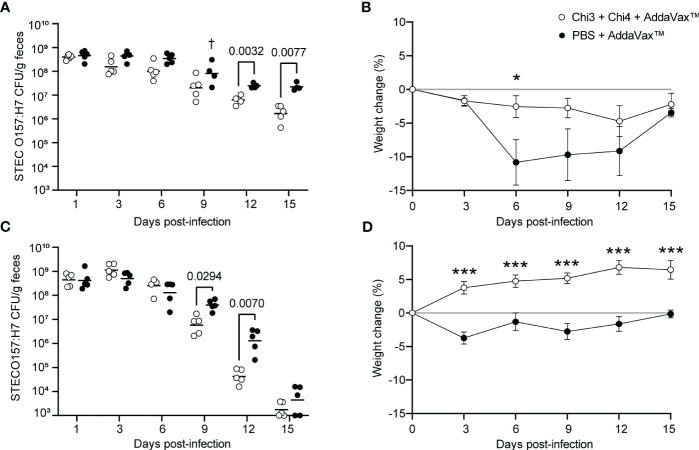
Protection against STEC O157:H7 conferred by immunization with chimeric proteins. **(A)** Five immunized mice and five control mice were orally inoculated with 10^9^ CFU of STEC O157:H7, following the protocol shown in **Figure 1B**. During the 15-day infection period, these mice were administered streptomycin in their drinking water. Data are shown as the number of CFU of the challenge strain per 1 g of feces. † Indicates an animal died. **(B)** Percentage of body weight change (± standard deviation) of the animals over a period of 15 days after infection with 10^9^ CFU of STEC O157:H7. **(C)** Five immunized mice and five control mice were orally inoculated with 10^6^ CFU of STEC O157:H7, as shown in **Figure 1C**. These mice were given streptomycin in their drinking water only two days before infection. **(D)** Percentage of body weight change (± standard deviation) of the animals over a period of 15 days after infection with 10^6^ CFU of STEC O157:H7. Statistical differences were determined by two-way ANOVA test followed by Sidak’s multiple comparison test. (* P < 0.05, ** P < 0.005, *** P < 0.0005).

The protection conferred by the vaccine formulation was evaluated by challenging mice with STEC O157:H7, following the protocol described in **Figure 1B**. Notably, immunized mice showed a lower fecal shedding of STEC O157:H7 compared to control mice, with significant differences obtained on days 12 and 15 after infection (**Figure 6A**). However, none of the mice achieved clearance of the challenge bacteria. It is important to note that on day 9, a control mouse showed lethargy, piloerection, anorexia, and eventually died. In addition, control mice exhibited a higher loss of weight than immunized mice (**Figure 6B**), which was particularly evident on day 6, where a statistically significant difference was observed. After day 9, control mice began to gain weight and on day 15, the percentage of weight change between both groups was practically the same.

To further ascertain the protection conferred by the vaccine formulation, we performed a second challenge experiment with a modified infection protocol, as described in **Figure 1C**. As a result, immunized mice showed a lower fecal shedding of STEC O157:H7 compared to control mice, with significant differences observed on days 9 and 12 after infection (**Figure 6C**). However, on day 15, the shedding levels of O157:H7 were similar between both groups, and in some mice, the bacteria were cleared and not detected in their feces. Moreover, it is noteworthy that immunized mice gained weight while control mice had slight weight loss, with significant differences observed between the two groups from day 3 onwards (**Figure 6D**). These results suggest that immunization with the vaccine formulation confers protection against STEC O157:H7.

**Figure 7:**
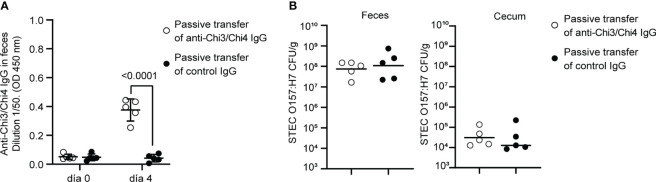
Protection against STEC O157:H7 conferred by passive immunization. **(A)** Passive immunization with anti-Chi3/Chi4 IgG. Five mice were passively immunized, through the tail vein, with two doses of 200 µg of anti-Chi3/Chi4 IgG, as shown in **Figure 1D**. As a control, five mice were administered 200 µg of IgG obtained from the serum of control mice (PBS + AddaVax™). The graph shows the presence of anti-Chi3/Chi4 IgG in feces on day 4 after antibody transfer. Statistical differences were determined by two-way ANOVA test, followed by Sidak’s multiple comparison test. **(B)** Fecal shedding and colonization of STEC O157:H7 in cecum in passively immunized animals. Five mice per group were orally inoculated with 10^6^ CFU of STEC O157:H7, following the protocol shown in **Figure 1D**. These mice were given streptomycin in their drinking water only two days before infection. On day 4 after infection, feces were collected, and the mice were euthanized to obtain the cecum. The levels of fecal shedding are indicated as the number of CFU of the challenge bacteria per gram of feces. The levels of colonization in the cecum are indicated as the number of CFU per gram of tissue. Statistical differences between groups were determined by the Mann-Whitney test.

Next, we sought to investigate the role of anti-Chi3/Chi4 IgG antibodies in protection against STEC O157:H7. For this, IgG was purified from sera of actively immunized and control mice. Subsequently, two groups of naïve mice were treated with streptomycin for two days and then subjected to passive transfer of anti-Chi3/Chi4 IgG or control IgG, as described in **Figure 1D**. Simultaneously, these mice were inoculated with a dose of 10^6^ CFU of STEC O157:H7. As shown in **Figure 7**, no reduction in the fecal shedding or colonization in the cecum of STEC O157:H7 were observed in mice receiving hyperimmune IgG compared to controls.

### Evaluation of the protection against STEC O91:H21

**Figure 8:**
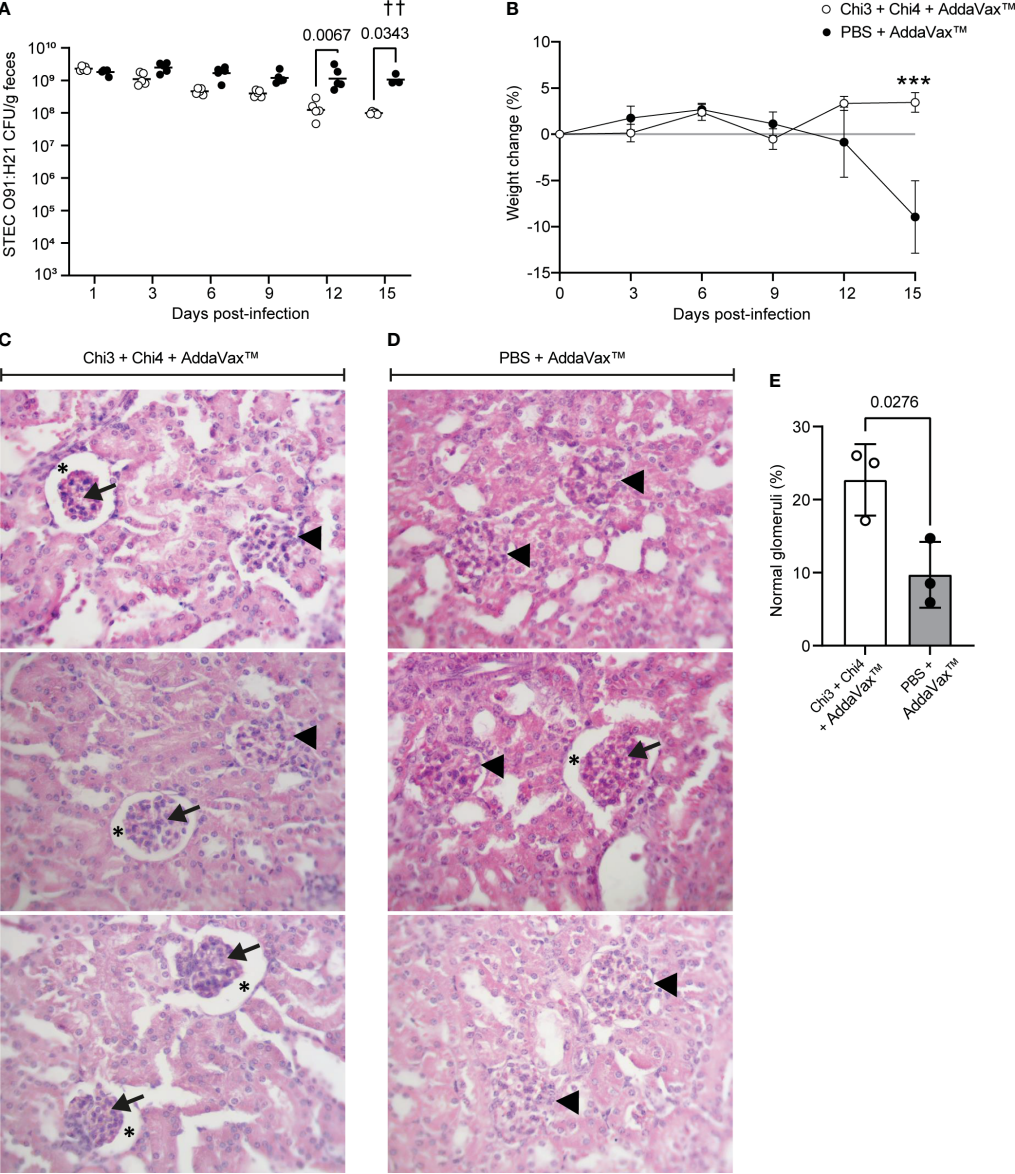
Protection against STEC O91:H21 conferred by immunization with chimeric proteins. **(A)** 5 mice per group were orally inoculated with 107 CFU of STEC O91:H21, following the protocol shown in **Figure 3B**. Data are shown as the number of CFU of the challenge strain per 1 g of feces. ^†^ Indicates deceased mouse. Statistical differences and P value were determined by two-way ANOVA test, followed by Sidak’s multiple comparison test. **(B)** Percentage of body weight change ( ± SD) of the animals during a 15-day period after infection. Statistical differences and P value were determined by two-way ANOVA test, followed by Sidak’s multiple comparison test (*** P < 0.0005). **(C, D)** Renal histology of the cortical zone of mice infected with O91:H21. At day 15 after infection, 3 animals per group were euthanized and their kidneys were processed for histological analysis. Glomeruli with an altered structure (arrowheads) were observed in both immunized and control mice. In addition, it was observed adhesion of the glomerular tuft to the Bowman’s capsule lamina. Arrows: Glomerular tuft. Asterisks: Urinary space of the renal corpuscle. Stain: Hematoxylin-Eosin. Magnification: 400X. **(E)** Percentage of non-affected glomeruli normalized by the total number of glomeruli observed in a histological section per animal. Differences between groups were determined by two-tailed Student’s t test. The symbol “†” indicates 1 deceased mouse. Therefore, the symbol “††” is shown in the figure indicating that 2 mice died.

We also evaluate the protection conferred by the vaccine formulation against the STEC O91:H21 strain V07-4-4. For this, immunized and control mice were orally inoculated as described in **Figure 1B**. As a result, immunized mice showed a lower fecal shedding of STEC O91:H21 compared to control mice, with significant differences observed on days 12 and 15 post-infection (**Figure 8A**). However, none of the mice achieved clearance of the challenge bacteria.

Furthermore, it is worth noting that control mice exhibited a decrease in weight starting from day 12, and this weight loss was statistically significant on day 15 when compared to immunized mice (**Figure 8B**). Importantly, it should be mentioned that two mice from the control group experienced health impairment characterized by a hunched posture on day 14 and died overnight on day 15.

To determine the renal involvement in these animals, a histological analysis was performed, revealing that both groups presented glomeruli with structural alterations (**Figures 8C, D**). However, the number of affected glomeruli was slightly lower in immunized mice (**Figure 8E**). Taken together, these results indicate that immunization with the vaccine formulation confers partial protection against STEC O91:H21.

### Characterization of the effector functions of anti-Chi3/Chi4 IgG in the immune response against STEC

An effective humoral response against enteric infections requires antibodies that can neutralize pathogens at the surface of the intestinal mucosa. Because we found that our vaccine formulation elicited a humoral response based mainly on IgG, we were interested in elucidating the effector functions of these antibodies.

**Figure 9:**
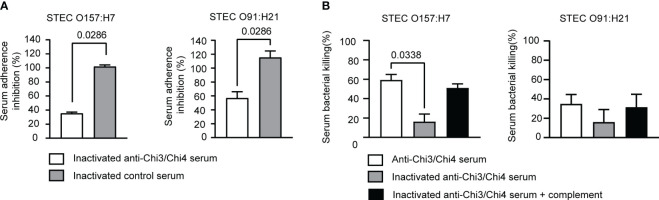
Determination of effector functions of anti-Chi3/Chi4 IgG. **(A)** Serum adherence inhibition assay. The serum from mice immunized with Chi3 + Chi4 + AddaVax™ partially inhibits the adhesion of STEC O157:H7 and STEC O91:H21 to Caco-2 intestinal epithelial cells. An inoculum of approximately 10^6^ CFU of bacteria was incubated in DMEM medium, or DMEM medium containing inactivated hyperimmune or control serum at a 1:5 dilution, for 30 minutes at 37°C with gentle agitation. After the incubation, the bacteria were used to infect Caco-2 cells, as described in material and methods. The graphs show the percentage of adherent bacteria in relation to the number of bacteria added, normalized with the percentage of adherent bacteria that were incubated in DMEM medium (without serum). The data correspond to two independent experiments performed in duplicate. Error bars represent standard deviation. Statistical differences between groups were determined using the Mann-Whitney test. **(B)** Serum bactericidal activity assay. An inoculum of approximately 10^6^ CFU of bacteria (STEC O157:H7 or STEC O91:H21) was incubated with hyperimmune or control sera at a dilution of 1:5, either active or inactivated, and inactivated sera supplemented with exogenous mouse complement. CFU counts were determined by plating on LB agar plates. Bacterial killing was normalized with the bacteria that survived after exposure to the respective control serum. The data correspond to two independent experiments performed in duplicate. Error bars represent standard deviation. Statistical differences between groups were determined using the Kruskal-Wallis test.

Therefore, it was determined whether hyperimmune serum was capable of inhibiting the adherence of the STEC O157:H7 and O91:H21 strains to Caco-2 cells. When used at a final concentration of 20%, hyperimmune serum inhibited the adhesion of O157:H7 and O91:H21 by approximately 60% and 50%, respectively (**Figure 9A**). In contrast, control serum was unable to inhibit the adhesion of these bacteria. This result supports a possible role for anti-Chi3/Chi4 IgG in inhibiting the adhesion of both bacteria to intestinal epithelial cells.

Finally, the bactericidal activity of hyperimmune serum against both bacteria was evaluated. As shown in **Figure 9B**, hyperimmune serum exhibited a bactericidal activity against STEC O157:H7 of approximately 50%. However, when the serum was heat-inactivated, the bactericidal activity decreased significantly to 25%. When exogenous complement was added, the bactericidal activity was partially restored. Therefore, the bactericidal activity of hyperimmune serum against O157:H7 is possibly associated with activation of the complement *via* the classical pathway.

When the bactericidal activity of the hyperimmune serum against O91:H21 was evaluated, it was found to be modest, reaching approximately 35%. Heat inactivation of the serum led to a decrease in bactericidal activity, although it was not statistically significant. Partial recovery was observed with the addition of exogenous complement, but again, it was not statistically significant. Therefore, this result shows that the hyperimmune serum exhibits a slight bactericidal activity against O91:H21.

## Discussion

In a previous study, we successfully developed a vaccine candidate against STEC based on the Chi1 and Chi2 proteins (28, 29). The design of these proteins involved the precise identification of epitopes through a peptide microarray assay. It is noteworthy that the epitope identification through this assay exhibited a strong correlation with the epitopes that were predicted using the algorithms available on the IEDB server. Building upon this knowledge, we have employed a similar bioinformatics approach in the current study to rationally design a novel chimeric protein-based vaccine against STEC.

One of the primary challenges in developing vaccines against STEC is the potential for disrupting the commensal gut microbiota. To address this concern, we carefully selected STEC antigens that are absent in commensal *E. coli* strains. By focusing on these specific antigens, we aim to minimize any potential damage to the beneficial gut microbiota while still targeting the virulence factors associated with STEC infection.

Vaccine development strategies against STEC have typically focused on LEE-encoded antigens, such as Tir, Intimin, and EspA (18–26). In our approach, we incorporate these three antigens and two outer membrane proteins, OmpT and Cah, which our group previously demonstrated as effective immunogens capable of eliciting protective immune responses against the pathogen (28, 29). Not all the virulent STEC strains carry the LEE PAI. For example, LEE-negative O91:H21 and O113:H21 are important STEC serotypes causing human disease (40, 62). OmpT and Cah are carried by these and other LEE-negative STEC strains (52), so incorporating these antigens into our vaccine formulation has the potential to provide wide-range of protective immunity to STEC. We also incorporate the AggA protein, which is the major subunit of the AAF/I fimbriae, an important virulence factor that mediate adherence of EAEC/STEC O104:H4 strains (63). Of note, AAF/I fimbriae have been shown to be immunogenic in mice (64). Furthermore, we include the Stx2B subunit since studies have shown that antibodies targeting this subunit can protect mice from the cytotoxic effects of Stx2 (65–67).

The *in silico* analysis of these proteins focused on predicting lineal B cell epitopes and MHC class II binding peptides, using several algorithms available on the IEDB server. Notably, the IEDB database has been enhanced with a vast collection of published epitopes and full-scale MHC-binding peptides, which has improved its predictive power (68). As a result, we predicted several epitopes, some of which are located consecutively or partially overlapping, suggesting the presence of antigenic domains. Based on these results, we rationally designed Chi3 and Chi4 fusion proteins, which incorporate the identified antigenic domains (**Table 1**). Production and purification of these proteins were successfully accomplished, and their antigenicity was confirmed using sera from patients who had developed HUS, as well as from cattle that were experimentally infected with STEC O157:H7 (**Figure 2**).

After confirming the antigenicity of Chi3 and Chi4, we proceeded to evaluate them as a vaccine candidate against STEC. For this, we formulated the chimeric proteins with the AddaVax™ adjuvant and evaluated their immunogenicity and protective efficacy in the streptomycin-treated mouse model.

The results showed that immunization with our vaccine formulation triggers a specific humoral and cellular immune response in mice. Specifically, we found significantly higher titers of specific IgG antibodies in both serum and feces of immunized mice compared to control mice. The systemic IgG response was characterized by the significant production of all IgG isotypes, while the intestinal mucosal IgG response was primarily of IgG1 antibodies. However, we did not observe any IgA responses elicited by this vaccine formulation (**Figure 3**). Additionally, our findings showed that immune sera recognized antigens present on the surface of O157:H7 as well as OMVs obtained from this bacterium (**Figure 4**). This finding suggests that the vaccine could potentially induce a response against OMVs, which play key roles in STEC pathogenesis (69, 70).

Regarding the cellular response, the results showed that immunized mice exhibited responses of antigen-specific CD4 T cells secreting IFNγ, IL-4, and IL-17 compared to the control group (**Figure 5**). While the IFNγ response evidenced the activation of Th1 cells, the increased production of IL-4 and IL-17 also suggests the involvement of Th2 and Th17 cells, respectively. Taken together, these results indicate that the vaccine formulation is capable of eliciting a robust IgG response and a mixed Th1-, Th2-, and Th17-like T cell response against STEC O157:H7.

Due to its abundance in the intestinal mucosa, IgA antibodies have been considered the main adaptive immune response against enteric infections. However, it has been demonstrated that some of these infections can be eliminated in the absence of IgA, indicating that other antibody responses are also important (71, 72). In contrast, the presence and effector function of IgG in the intestinal mucosa has largely been ignored in the literature (73).

The murine pathogen *C. rodentium* has been widely used to characterize the immune response against A/E pathogens. One of the first studies to highlight the role of IgG against this bacterium was conducted by Ghaem-Maghami et al. (74). In this study, subcutaneous immunization of mice with the Intimin protein generated a strong humoral immune response characterized by specific IgG that prevented colonization by *C. rodentium*. A subsequent study showed that the elimination of *C. rodentium* and the survival of infected mice were dependent on B lymphocytes and IgG but not on the production secretory IgA or IgM (75). More recently, Kamada et al. (76) described one of the mechanisms by which IgG directs the elimination of *C. rodentium*. During mouse infection, two populations of *C. rodentium* reside in the intestine; a phenotypically “virulent” population that expresses LEE adheres to the colon mucosa, and an “avirulent” population that does not express LEE is displaced towards the lumen. As a result of infection, specific IgG antibodies are generated mainly against virulence factors encoded in LEE. These antibodies are transported to the intestinal lumen, presumably through the neonatal Fc receptor (FcRn), where they bind to virulent bacteria expressing LEE, leading to their elimination by neutrophils that migrate through the epithelium. In contrast, the “avirulent” *C. rodentium* population is not opsonized by IgG but is eventually excluded by the microbiota.

Regarding STEC, it has been reported that vaccine candidates based on proteins encoded in LEE (Tir, Intimin, EspA) and on Stx2B induce specific IgG antibodies that reduce the shedding of this pathogen in mice (18, 20, 27, 77). In agreement with these studies, we found that our vaccine formulation conferred partial protection against O157:H7. Specifically, we observed that immunized mice exhibited lower fecal shedding of STEC O157:H7 and had higher weight gain compared to control mice (**Figures 6A–D**). When mice received streptomycin throughout the experiments, none of the immunized mice completely eliminate the challenge bacteria. However, when streptomycin was given only before infection, some immunized and control mice achieved clearance of the bacteria. Consequently, the absence of streptomycin may have allowed for recovery of the intestinal microbiota, ultimately facilitating effective clearance of the O157:H7 strain in both groups.

In passive immunized mice, protection against colonization by STEC O157:H7 was not observed (**Figure 7**). This result does not rule out the role of IgG in protection against this pathogen. However, it suggests that an effective immune response involves multiple arms of the immune system. Additionally, it is possible that the amount of transferred IgG was not sufficient to decrease intestinal colonization of STEC O157:H7.

In streptomycin-treated mice, it has been demonstrated that Stx2d-producing *E. coli*, such as the O91:H21 strain V07-4-4, can cause kidney damage and, in some cases, even lead to death (28, 62). Therefore, we also evaluate the protection conferred by the vaccine formulation against this STEC strain. As result, we also observed reduce shedding of STEC O91:H21 in immunize mice compared to control mice (**Figure 8A**). Importantly, two mice from the control group exhibited a compromised state of health and died on day 15, while none of the immunized mice died. Histological analysis revealed that both groups had glomeruli with structural alterations, but the number of affected glomeruli was slightly lower in immunized mice (**Figures 8C, D**). These findings suggest that the vaccine formulation may be effective in reducing the severity of STEC O91:H21 infection, but further studies are needed to optimize the vaccine and enhance its protective efficacy.

We also investigated the effector functions of the IgG antibodies elicited by the vaccine formulation. We found that immune sera significantly inhibit the adherence of both O157:H7 and O91:H21 strains to Caco-2 cells, with a greater inhibition observed for O157:H7 (**Figure 9A**). In addition, the bactericidal activity of the immune serum against O157:H7 was found to be approximately 50%, which was reduced when the serum was heat inactivated. The addition of exogenous complement partially restored the bactericidal activity (**Figure 9B**), suggesting that the classical complement pathway may be involved in the bactericidal activity. These results, along with the data from the challenge experiments, suggest that IgG responses are involved in the protection against STEC. The role of individual IgG isotypes in mediating the protection against STEC was not determined in this study. Future investigations will address this.

Our findings have important implications for the design of effective vaccines against STEC, as they suggest that IgG responses are involved in the control of these infections. However, it is important to mention that our results do not rule out that other classes of antibodies also participate in the protection against this pathogen. Overall, the results demonstrated the immunogenicity and protective responses elicited by our vaccine candidate against STEC. This study provides a starting point for the optimization of these chimeric proteins as a vaccine candidate against STEC. Future experiments will evaluate the formulation of Chi3 and Chi4 proteins, along with other adjuvants and routes of administration. Additionally, we will evaluate the immune response against other strains such the EAEC/STEC O104:H4, as our vaccine formulation includes the AggA protein.

## Data availability statement

The original contributions presented in the study are included in the article/**Supplementary Materials**, further inquiries can be directed to the corresponding authors.

## Ethics statement

The animal study was reviewed and approved by Comité Institucional de Cuidado y Uso de Animales (CICUA) (Protocol 19250-ODO-UCH), Universidad de Chile.

## Author contributions

Conceptualization and experimental design, DM, RV, and LC. *In vivo* experiments, DM, RG-B, PP, DS, CV, LG, and AO. *In vitro* experiments, DM, RG-B, PP, and LG. Histological analysis, HM. Data interpretation and writing the manuscript, DM and LC. Review and edition, LC and RV All authors contributed to the article and approved the submitted version.

## In memoriam

In memory of Carmenza Forero (1955 – 2020), beloved mother and best teacher.
