# Supplementary material for: A chimeric protein-based vaccine elicits a strong IgG antibody response and confers partial protection against Shiga toxin-producing Escherichia coli in mice
Source: Front Immunol. 2023 Jul 27;14:1186368. doi: 10.3389/fimmu.2023.1186368 (PMC10413102; doi:10.3389/fimmu.2023.1186368)
Supplement: Supplementary file 1 [file DataSheet_1.pdf]

Supplementary information

A chimeric protein-based vaccine elicits a strong IgG antibody response and confers partial protection against Shiga toxin-producing *Escherichia coli* in mice

David A. Montero[1,2,3]*, Richard Garcia-Betancourt[1,2], Roberto M. Vidal[2,4], Juliana Velasco[5,6], Pablo A. Palacios[1,2], Daniela Schneider[1,2], Carolina Vega[7], Leonardo Gómez[3], Hernán Montecinos[8], Rodrigo Soto-Shara[3], Ángel Oñate[3], Leandro J. Carreño[1,2]*

[1] Programa de Inmunología, Instituto de Ciencias Biomédicas, Facultad de Medicina, Universidad de Chile, Santiago, Chile.
[2] Instituto Milenio de Inmunología e Inmunoterapia, Facultad de Medicina, Universidad de Chile, Santiago, Chile.
[3] Departamento de Microbiología, Facultad de Ciencias Biológicas, Universidad de Concepción, Concepción, Chile.
[4] Programa de Microbiología y Micología, Instituto de Ciencias Biomédicas, Facultad de Medicina, Universidad de Chile, Santiago, Chile.
[5] Unidad de Paciente Crítico, Clínica Hospital del Profesor, Santiago, Chile.
[6] Programa de Formación de Especialista en Medicina de Urgencia, Universidad Andrés Bello, Santiago, Chile
[7] Plataforma Experimental, Facultad de Odontología, Universidad de Chile, Santiago, Chile.
[8] Departamento de Biología Celular, Facultad de Ciencias Biológicas, Universidad de Concepción, Concepción, Chile.

* Corresponding authors. Correspondence should be addressed to David A. Montero (davmontero@udec.cl) and Leandro J. Carreño (email: leandrocarreno@uchile.cl)

Supplementary Table 1. Laboratory animal supervision protocol*

Variable	Ranges	Score
**Weight Loss (0 to 3)**	● Normal (no weight loss or animal grows normally).<br>● Weight loss less than 10%.<br>● Weight loss between 10% and 20%. Possible alteration in the appearance or amount of feces.<br>● Weight loss greater than 20%, the animal does not consume water or food.	0<br>1<br>2<br>3
**Appearance (0 to 3)**	● Normal.<br>● Poor coat condition.<br>● Poor coat condition and/or presence of ocular or nasal secretions.<br>● Abnormal posture.	0<br>1<br>2<br>3
**Spontaneous Behavior (0 to 3)**	● Normal.<br>● Small changes.<br>● Inactivity<br>● Self-mutilation, abnormal vocalization, very restless or immobile animals.	0<br>1<br>2<br>3
**Behavior in Response to Handling (0 to 3)**	● Normal.<br>● Small changes.<br>● Moderate changes<br>● Aggressive or comatose animals.	0<br>1<br>2<br>3
**Pathologies caused by STEC (0 to 3)**	● None.<br>● Diarrhea.<br>● Severe diarrhea.<br>● Severe diarrhea and lethargy.	0<br>1<br>2<br>3
**Total Score**		

Note: when an animal scores a 3 on more than one parameter, all 3's become 4.

The suggested corrective measures based on the score obtained for each animal are as follows:

Score:

From 0-5 | Normal

From 5-10 | Monitor carefully

From 10-15 | Euthanize

* Modified Animal Supervision Protocol from the protocol proposed by Morton and Griffiths (1985) (Vet Rec. 1985, 116:431–436. doi: 10.1136/vr.116.16.431).

Supplementary Table 2. Antigenic proteins of STEC that were used to design Chimera 3 and Chimera 4 proteins.

**Protein**	**Biological Function**	**Immunogenic evaluation in mice**	**Reference**
Stx2B (subunit B)	The pentameric B subunit is recognized by the eukaryotic receptor globotriaosylceramide, allowing internalization of Shiga toxin	Induction of IgG. Protection against lethal dose.	(1,2)
Intimin	Adhesion	Induction of IgG responses. Reduces shedding of STEC	(3–6)
Tir	Translocated intimin receptor. Adhesion.	Induction of IgG responses. Reduces shedding of STEC	(3,5,7)
EspA	SST3 structural protein	Induction of IgG responses. Reduces shedding of STEC	(3,5)
OmpT	Protease. Degrades the antimicrobial peptide LL-37. Participates in the outer membrane vesicle biogenesis	Induction of IgG responses. Reduces shedding of STEC	(8–10)
Cah	Adhesion, autoaggregation	Induction of IgG. Reduces colonization of EHEC	(10)
AggA	AAF/I, Protein of Aggregative Adherence Factor I. Adhesion, autoaggregation	Induction of IgG.	(11)

References:

1. Ludwig K, Karmali MA, Sarkim V, Bobrowski C, Petric M, Karch H, Mu DE, Pa R. Antibody Response to Shiga Toxins Stx2 and Stx1 in Children with Enteropathic Hemolytic-Uremic Syndrome. (2001) 39:2272–2279. doi: 10.1128/JCM.39.6.2272

2. Gao X, Cai K, Shi J, Liu H, Hou X, Tu W, Xiao L, Wang Q, Wang H. Immunogenicity of a novel Stx2B-Stx1B fusion protein in a mice model of Enterohemorrhagic Escherichia coli O157:H7 infection. *Vaccine* (2009) 27:2070–2076. doi: 10.1016/j.vaccine.2009.01.115

3. Asper DJ, Karmali MA, Townsend H, Rogan D, Potter AA. Serological response of shiga toxin-producing Escherichia coli type III secreted proteins in sera from vaccinated rabbits, naturally infected cattle, and humans. *Clin Vaccine Immunol* (2011) 18:1052–1057. doi: 10.1128/CVI.00068-11

4. Gao X, Cai K, Li T, Wang Q, Hou X, Tian R, Liu H, Tu W, Xiao L, Fang L, et al. Novel fusion protein protects against adherence and toxicity of enterohemorrhagic Escherichia coli O157:H7 in mice. *Vaccine* (2011) 29:6656–63. doi: 10.1016/j.vaccine.2011.06.106

5. Amani J, Salmanian AH, Rafati S, Mousavi SL. Immunogenic properties of chimeric protein from espA, eae and tir genes of Escherichia coli O157:H7. *Vaccine* (2010) 28:6923–6929. doi: 10.1016/j.vaccine.2010.07.061

6. Cataldi A, Yevsa T, Vilte D a, Schulze K, Castro-Parodi M, Larzábal M, Ibarra C, Mercado EC, Guzmán C a. Efficient immune responses against Intimin and EspB of enterohaemorragic Escherichia coli after intranasal vaccination using the TLR2/6 agonist MALP-2 as adjuvant. *Vaccine* (2008) 26:5662–7. doi: 10.1016/j.vaccine.2008.07.027

7. Babiuk S, Asper DJ, Rogan D, Mutwiri GK, Potter A a. Subcutaneous and intranasal immunization with type III secreted proteins can prevent colonization and shedding of Escherichia coli O157:H7 in mice. *Microb Pathog* (2008) 45:7–11. doi: 10.1016/j.micpath.2008.01.005

8. Montero D, Orellana P, Gutiérrez D, Araya D, Salazar JC, Prado V, Oñate A, Del Canto F, Vidal R. Immunoproteomic analysis to identify Shiga toxin-producing Escherichia coli outer membrane proteins expressed during human infection. *Infect Immun* (2014) 82:4767–77. doi: 10.1128/IAI.02030-14

9. Premjani V, Tilley D, Gruenheid S, Le Moual H, Samis J a. Enterohemorrhagic Escherichia coli OmpT regulates outer membrane vesicle biogenesis. *FEMS Microbiol Lett* (2014) doi: 10.1111/1574-6968.12463

10. Montero DA, Del Canto F, Salazar JC, Céspedes S, Cádiz L, Arenas-Salinas M, Reyes J, Oñate Á, Vidal RM. Immunization of mice with chimeric antigens displaying selected epitopes confers protection against intestinal colonization and renal damage caused by Shiga toxin-producing Escherichia coli. *npj Vaccines* (2020) 5:20. doi: 10.1038/s41541-020-0168-7

11. Bouzari S, Dashti A, Jafari A, Oloomi M. Immune response against adhesins of enteroaggregative Escherichia coli immunized by three different vaccination strategies (DNA/DNA, Protein/Protein, and DNA/Protein) in mice. *Comp Immunol Microbiol Infect Dis* (2010) 33:215–225. doi: 10.1016/j.cimid.2008.10.002

Supplementary Table 3. Prediction of potential linear B-cell epitopes and MCH-II binding peptides in the Stx2B subunit (Sequence ID: EFA6422208.1)

Amino acid sequence:

******* ********* ********

ADCAKGKIEFSKYNENDTFTVKVAGKEYWTSRWNLQPLLQSAQLTGMTVTIKSSTCESGSGFAEVQFNND

B-cell epitopes predicted by BepiPred 2.0[1]

Epitope ID	Sequence	Length
Stx2B-B1	IEFSKYNENDTFT	11
Stx2B-B2	KEYWTSRWNLQPLLQSAQLT	20
Stx2B-B3	STCESGSGFAE	11

B-cell epitopes predicted by Kolaskar and Tongaonker antigenicity method[2]

Epitope ID	Sequence	Length
Stx2B-K1	TVKVAGK	7
Stx2B-K2	LQPLLQSAQ	9
Stx2B-K3	TIKSSTCE	8

MHC-II binding peptides by NetMHCIIpan 4.1[3]

Core Peptide ID	Allele	Core sequence	Peptide sequence	Score
Stx2B-CP1	HLA-DRB3*02:02	YNENDTFTV	FSKYNENDTFTVKVA	0.6982
Stx2B-CP1	HLA-DRB3*02:02	YNENDTFTV	EFSKYNENDTFTVKV	0.6031
Stx2B-CP1	HLA-DRB3*02:02	YNENDTFTV	SKYNENDTFTVKVAG	0.5393

[1] A threshold value of 0.5 was used. Predicted epitopes are shown underlined in the amino acid sequence.
[2] A threshold value of 1.0 and window of 7 were used. Predicted epitopes are indicated with asterisk in the amino acid sequence.
[3] A set of seven human HLA-DR alleles were analyzed: HLA-DRB1*03:01, HLA-DRB1*07:01, HLA-DRB1*15:01, HLA-DRB3*01:01, HLA-DRB3*02:02, HLA-DRB4*01:01, HLA-DRB5*01:01. An epitope length of 15 was defined as the default parameter. Binding peptides with a score > 0.5 were selected and are shown in yellow in the amino acid sequence.

Supplementary Table 4. Prediction of potential lineal B-cell epitopes, and MCH-II binding peptides in the Tir protein (Sequence ID: WP_001301454.1)

Amino acid sequence:

******** ********* *******
MPIGNLGHNPNVNNSIPPAPPLPSQTDGAGGRGQLINSTGPLGSRALFTPVRNSMADSGDNRASDVPGLP

**** ********* ******* ********* ***
VNPMRLAASEITLNDGFEVLHDHGPLDTLNRQIGSSVFRVETQEDGKHIAVGQRNGVETSVVLSDQEYAR

**** ********* *********
LQSIDPEGKDKFVFTGGRGGAGHAMVTVASDITEARQRILELLEPKGTGESKGAGESKGVGELRESNSGA

****
ENTTETQTSTSTSSLRSDPKLWLALGTVATGLIGLAATGIVQALALTPEPDSPTTTDPDAAASATETATR

****
DQLTKEAFQNPDNQKVNIDELGNAIPSGVLKDDVVANIEEQAKAAGEEAKQQAIENNAQAQKKYDEQQAK

******* **************************
RQEELKVSSGAGYGLSGALILGGGIGVAVTAALHRKNQPVEQTTTTTTTTTTTSARTVENKPANNTPAQG

********************** *****
NVDTPGSEDTMESRRSSMASTSSTFFDTSSIGTVQNPYADVKTSLHDSQVPTSNSNTSVQNMGNTDSVVY

****** **************
STIQHPPRDTTDNGARLLGNPSAGIQSTYARLALSGGLRHDMGGLTGGSNSAVNTSNNPPAPGSHRFV

B-cell epitopes predicted by BepiPred 2.0[1]

Epitope ID	Sequence	Length
Tir-B1	NLGHNPNVNNSIPPAPPLPSQTDGAGGRGQLI NSTGPLGSRA	42
Tir-B2	RNSMADSGDNRASDVPGLPVNPMRL	25
Tir-E3	EVLHDHGPLDTLNRQIGSS	19
Tir-E4	TQEDGKHIAVGQRNGVE	17
Tir-E5	DQEYARLQSIDPEG	14
Tir-E6	LELLEPKGTGESKGAGESKGVGELRESNSGAENTT ETQTSTSTSSLRSD	49
Tir-E7	PEPDSPTTTDPDAAASATETATRDQLTKEAFQNPD NQKVNIDELGNAIPSGVLKD	55
Tir-E8	KAAGEEAKQQAIENNAQAQKKYDEQQAKRQEELKV SSGAGY	41
Tir-E9	RKNQPVEQTT	10
Tir-E10	RTVENKPANNTPAQGNVDTPGSEDTMESRRSSMAS TSSTFFDTSSIGTVQNPYADVKTSLHDSQVPTSNS NTSVQNMGNTD	81
Tir-E11	PRDTTDNGARL	11
Tir-E12	GGLTGGSNSAVNTSNNPPAPGSH	23

B-cell epitopes predicted by Kolaskar and Tongaonker antigenicity method[2]

Epitope ID	Sequence	Length
Tir-K1	PPAPPLPS	8
Tir-K2	GSRALFTPV	9
Tir-K3	SDVPGLPVNPM	11
Tir-K4	GFEVLHDHG	9
Tir-K5	GSSVFRV	7
Tir-K6	ETSVVLSDQ	9
Tir-K7	YARLQSI	7
Tir-K8	HAMVTVASD	9
Tir-K9	QRILELLEP	9

Tir-K10		KLWLALGTVATGLIGLAATGIVQALALTPE		30
Tir-K11		PSGVLKDDVVANI		13
Tir-K12		ELKVSSG		7
Tir-K13		GYGLSGALILGGGIGVAVTAALHRKN		26
Tir-K14		IGTVQNPYADVKTSLHDSQVPT		22
Tir-K15		DSVVYSTIQHP		11
Tir-K16		GIQSTYARLALSGG		14
**MHC-II binding peptides by NetMHCIIpan 4.1[3]**				
Core Peptide ID	Allele	Core sequence	Peptide sequence	Score
Tir-CP1	HLA-DRB1*03:01	VASDITEAR	MVTVASDITEARQRI	0.9442
Tir-CP1	HLA-DRB1*03:01	VASDITEAR	AMVTVASDITEARQR	0.9442
Tir-CP2	HLA-DRB1*03:01	LRSDPKLWL	TSSLRSDPKLWLALG	0.9382
Tir-CP2	HLA-DRB3*02:02	LRSDPKLWL	STSSLRSDPKLWLAL	0.9138
Tir-CP2	HLA-DRB5*01:01	LRSDPKLWL	TSTSSLRSDPKLWLA	0.8994
Tir-CP1	HLA-DRB1*07:01	VASDITEAR	VTVASDITEARQRIL	0.8877
Tir-CP1	HLA-DRB3*02:02	VASDITEAR	HAMVTVASDITEARQ	0.8845
Tir-CP3	HLA-DRB5*01:01	YGLSGALIL	GAGYGLSGALILGGG	0.8843
Tir-CP4	HLA-DRB1*03:01	VLSDQEYAR	TSVVLSDQEYARLQS	0.8637
Tir-CP3	HLA-DRB4*01:01	YGLSGALIL	SGAGYGLSGALILGG	0.8439
Tir-CP4	HLA-DRB3*02:02	VLSDQEYAR	ETSVVLSDQEYARLQ	0.8363
Tir-CP2	HLA-DRB1*07:01	LRSDPKLWL	SSLRSDPKLWLALGT	0.8184
Tir-CP5	HLA-DRB1*03:01	LLGNPSAGI	GARLLGNPSAGIQST	0.8042
Tir-CP6	HLA-DRB1*03:01	VAVTAALHR	GIGVAVTAALHRKNQ	0.7797
Tir-CP5	HLA-DRB1*03:01	LLGNPSAGI	NGARLLGNPSAGIQS	0.7560

[1] A threshold value of 0.5 was used. Predicted epitopes are shown underlined in the amino acid sequence.
[2] A threshold value of 1.0 and window of 7 were used. Predicted epitopes are indicated with asterisk in the amino acid sequence.
[3] A set of seven human HLA-DR alleles were analyzed: HLA-DRB1*03:01, HLA-DRB1*07:01, HLA-DRB1*15:01, HLA-DRB3*01:01, HLA-DRB3*02:02, HLA-DRB4*01:01, HLA-DRB5*01:01. An epitope length of 15 was defined as the default parameter. Binding peptides with a score > 0.75 were selected and are shown in yellow in the amino acid sequence.

Supplementary Table 5. Prediction of potential lineal B-cell epitopes and MCH-II binding peptides in the AggA protein (Sequence ID: EFB7498699.1)

Amino acid sequence:

```
   *******                  *********                       ********
ITLGLVSLLSGGANAASQQTTQTIRLTVTNDCPVTITTTPPQTVGVSSTTPIGFSAKVTTSDQCIKAGAK

          *************                                 ******* ******
VWLWGTGPANKWVLQHAKVAKQKYTLNPSIDGGADFVNQGTDAKIYKKLTSGNKFLNASVSVNPKTQVLI

**
PGEYTMILHAAVDF
```

B-cell epitopes predicted by BepiPred 2.0[1]

Epitope ID	Sequence	Length
AggA-B1	LSGGANAASQQ	11
AggA-B2	TTPPQTVGVSSTTPIG	16
AggA-B3	LQHAKVAKQKYTLNPSIDGGADFVNQGTDAK IYKKLTSGNKFL	43

B-cell epitopes predicted by Kolaskar and Tongaonker antigenicity method[2]

Epitope ID	Sequence	Length
AggA-K1	GLVSLLS	7
AggA-K2	TNDCPVTIT	9
AggA-K3	SDQCIKAG	8
AggA-K4	KWVLQHAKVAKQK	13
AggA-K5	NASVSVN	7
AggA-K6	KTQVLIPG	8

MHC-II binding peptides by NetMHCIIpan 4.1[3]

Core Peptide ID	Allele	Core sequence	Peptide sequence	Score
AggA-CP1	HLA-DRB3*02:02	YTLNPSIDG	KQKYTLNPSIDGGAD	0.9733
AggA-CP1	HLA-DRB3*02:02	YTLNPSIDG	AKQKYTLNPSIDGGA	0.9627
AggA-CP1	HLA-DRB3*02:02	YTLNPSIDG	VAKQKYTLNPSIDGG	0.9236
AggA-CP1	HLA-DRB3*02:02	YTLNPSIDG	QKYTLNPSIDGGADF	0.9021
AggA-CP2	HLA-DRB5*01:01	YKKLTSGNK	AKIYKKLTSGNKFLN	0.7905

[1] A threshold value of 0.5 was used. Predicted epitopes are shown underlined in the amino acid sequence.
[2] A threshold value of 1.0 and window of 7 were used. Predicted epitopes are indicated with asterisk in the amino acid sequence.
[3] A set of seven human HLA-DR alleles were analyzed: HLA-DRB1*03:01, HLA-DRB1*07:01, HLA-DRB1*15:01, HLA-DRB3*01:01, HLA-DRB3*02:02, HLA-DRB4*01:01, HLA-DRB5*01:01. An epitope length of 15 was defined as the default parameter. Binding peptides with a score > 0.75 were selected and are shown in yellow in the amino acid sequence.

Supplementary Table 6. Prediction of potential lineal B-cell epitopes and MCH-II binding peptides in the C-terminal 282-residue fragment of Intimin protein (Sequence ID: WP_000627885.1)

Amino acid sequence:

```
                                        ********                                    **
FDQTKASITEIKADKTTAVANGKDAIKYTVKVMKNGQPVNNQSVTFSTNFGMFNGKSQTQATTGNDGRATI

*****    *********    **********                        ************
TLTSSSAGKATVSATVSDGAEVKATEVTFFDELKIDNKVDIIGNNVRGELPNIWLQYGQFKLKASGGDGTY

        **********     ********      ***********************************
SWYSENTSIATVDASGKVTLNGKGSVVIKATSGDKQTVSYTIKAPSYMIKVDKQAYYADAMSICKNLLPST

*******                                 ***********
QTVLSDIYDSWGAANKYSHYSSMNSITAWIKQTSSEQRSGVSSTYNLITQNPLPGVNVNTPNVYAVCVE
```

B-cell epitopes predicted by BepiPred 2.0[1]

Epitope ID	Sequence	Length
Int-B1	GMFNGKSQTQAT	12
Int-B2	GAEVKATEV	9
Int-B3	IGNNVRGELPNIWLQYGQFKLKASGGDGT YSWYSENTS	38
Int-B4	KQAYYADAMSICKNLLPSTQT	21
Int-B5	DSWGAANKYSHYSSMNSITA	20
Int-B6	QTSSEQRSGV	10
Int-B7	PLPGVNVNTPNV	12

B-cell epitopes predicted by Kolaskar and Tongaonker antigenicity method[2]

Epitope ID	Sequence	Length
Int-K1	IKYTVKVM	8
Int-K2	TITLTSS	7
Int-K3	KATVSATVS	9
Int-K4	EVKATEVTFF	10
Int-K5	NIWLQYGQFKLK	12
Int-K6	IATVDASGKV	10
Int-K7	KGSVVIKA	8
Int-K8	TVSYTIKAPSYMIKVDKQAYYADAMSICKN L LPSTQTVLSDI	42
Int-K9	RSGVSSTYNLI	11

MHC-II binding peptides by NetMHCIIpan 4.1[3]

Core Peptide ID	Allele	Core sequence	Peptide sequence	Score
Int-CP1	HLA-DRB1*03:01	IKVDKQAYY	SYMIKVDKQAYYADA	0.9725
Int-CP1	HLA-DRB1*03:01	IKVDKQAYY	PSYMIKVDKQAYYAD	0.9668
Int-CP2	HLA-DRB1*03:01	LKIDNKVDI	FDELKIDNKVDIIGN	0.9621
Int-CP3	HLA-DRB1*03:01	IKADKTTAV	ITEIKADKTTAVANG	0.9547
Int-CP2	HLA-DRB1*03:01	LKIDNKVDI	FFDELKIDNKVDIIG	0.9498
Int-CP3	HLA-DRB1*03:01	IKADKTTAV	SITEIKADKTTAVAN	0.9424
Int-CP2	HLA-DRB1*03:01	LKIDNKVDI	DELKIDNKVDIIGNN	0.9414
Int-CP3	HLA-DRB3*01:01	IKADKTTAV	ITEIKADKTTAVANG	0.9363
Int-CP1	HLA-DRB1*03:01	IKVDKQAYY	YMIKVDKQAYYADAM	0.9332
Int-CP1	HLA-DRB1*03:01	IKVDKQAYY	APSYMIKVDKQAYYA	0.9279
Int-CP3	HLA-DRB3*01:01	IKADKTTAV	SITEIKADKTTAVAN	0.9250
Int-CP3	HLA-DRB1*03:01	IKADKTTAV	TEIKADKTTAVANGK	0.9132
Int-CP2	HLA-DRB1*03:01	LKIDNKVDI	TFFDELKIDNKVDII	0.8990
Int-CP3	HLA-DRB1*03:01	IKADKTTAV	ASITEIKADKTTAVA	0.8959

Int-CP3	HLA-DRB3*01:01	IKADKTTAV	ASITEIKADKTTAVA	0.8759
Int-CP4	HLA-DRB1*07:01	YNLITQNPL	SSTYNLITQNPLPGV	0.8687
Int-CP4	HLA-DRB1*07:01	YNLITQNPL	VSSTYNLITQNPLPG	0.8631
Int-CP3	HLA-DRB3*01:01	IKADKTTAV	TEIKADKTTAVANGK	0.8605
Int-CP1	HLA-DRB3*01:01	IKVDKQAYY	SYMIKVDKQAYYADA	0.7930
Int-CP5	HLA-DRB1*15:01	VTFFDELKI	ATEVTFFDELKIDNK	0.7876
Int-CP4	HLA-DRB1*07:01	YNLITQNPL	GVSSTYNLITQNPLP	0.7707

[1] A threshold value of 0.5 was used. Predicted epitopes are shown underlined in the amino acid sequence.
[2] A threshold value of 1.0 and window of 7 were used. Predicted epitopes are indicated with asterisk in the amino acid sequence.
[3] A set of seven human HLA-DR alleles were analyzed: HLA-DRB1*03:01, HLA-DRB1*07:01, HLA-DRB1*15:01, HLA-DRB3*01:01, HLA-DRB3*02:02, HLA-DRB4*01:01, HLA-DRB5*01:01. An epitope length of 15 was defined as the default parameter. Binding peptides with a score > 0.75 were selected and are shown in yellow in the amino acid sequence.

Supplementary Table 7. Prediction of potential lineal B-cell epitopes and MCH-II binding peptides in the passenger domain of the Cah protein (Sequence ID: AAG55356.1)

Amino acid sequence:

****

ADKVVQAGETVNDGTLTNHDNQIVFGTANGMTISTGLELGPDSEENTGGQWIQNGGIAGNTTVTTNGRQVV

*** ******** *******

LEGGTASDTVIRDGGGQSLNGLAVNTTLNNRGEQWVHEGGVATGTIINRDGYQSVKSGGLATGTIINTGAE

******* ******* ******** ******* *********

GGPDSDNSYTGQKVQGTAESTTINKNGRQIILFSGLARDTLIYAGGDQSVHGRALNTTLNGGYQYVHRDGL

******* ******* ************

ALNTVINEGGWQVVKAGGAAGNTTINQNGELRVHAGGEATAVTQNTGGALVTSTAATVIGTNRLGNFTVEN

******** ******** ******* ******* *******

GKADGVVLESGGRLDVLESHSAQNTLVDDGGTLAVSAGGKATSVTITSGGALIADSGATVEGTNASGKFSI

******* ******** ********

DGTSGQASGLLLENGGSFTVNAGGQAGNTTVGHRGTLTLAAGGSLSGRTQLSKGASMVLNGDVVSTGDIVN

AGEIR

B-cell epitopes predicted by BepiPred 2.0[1]

Epitope ID	Sequence	Length
Cah-B1	MTISTGLELGPDSEENTGGQWIQNGGIA	28
Cah-B2	TNGRQVVLEGGTASDTVIRDGGGQSLNGLAVN	32
Cah-B3	NNRGEQWVHEGGVATGTIINRDGYQSVKSGGLA	33
Cah-B4	NTGAEGGPDSDNSYTGQKVQGTAESTTINKNGRQIIL	37
Cah-B5	AGGDQSVHGRALNTTLNGGYQYVHRDGLA	29
Cah-B6	NEGGWQVVKAGGAAGNTTINQNGELRVHAGGEATAVTQ NTGGALVTSTAATVIGTNRLGNFTVENGKADGV	71
Cah-B7	GGRLDVLESHSA	12
Cah-B8	NTLVDDGGTL	10
Cah-B9	SGGALIADSGATVE	14
Cah-B10	NASGKFSIDGTSGQASG	17
Cah-B11	SFTVNAGGQAGNTTVGHRGT	20
Cah-B12	LSGRTQLSKGASMVLNGDVVSTGDIVNA	28

B-cell epitopes predicted by Kolaskar and Tongaonker antigenicity method[2]

Epitope ID	Sequence	Length
Cah-K1	RQVVLEG	7
Cah-K2	LNGLAVNT	8
Cah-K3	YQSVKSG	7
Cah-K4	GQKVQGT	7
Cah-K5	QIILFSGL	8
Cah-K6	RDTLIYAG	8
Cah-K7	DQSVHGR	7
Cah-K8	GYQYVHRDG	9
Cah-K9	WQVVKAG	7
Cah-K10	ELRVHAG	7
Cah-K11	GALVTSTAATVI	12
Cah-K12	DGVVLES	7
Cah-K13	RLDVLESH	8
Cah-K14	TLAVSAG	7
Cah-K15	ATSVTIT	7

Cah-K16		GGALIAD		7
Cah-K17		ASGLLLE		7
Cah-K18		TLTLAAGG		8
Cah-K19		NGDVVSTG		8
**MHC-II binding peptides by NetMHCIIpan 4.1**[3]				
Core Peptide ID	Allele	Core sequence	Peptide sequence	Score
Cah-CP1	HLA-DRB1*07:01	YQSVKSGGL	RDGYQSVKSGGLATG	0.9549
Cah-CP1	HLA-DRB1*07:01	YQSVKSGGL	NRDGYQSVKSGGLAT	0.9455
Cah-CP2	HLA-DRB3*02:02	FTVNAGGQA	GGSFTVNAGGQAGNT	0.9433
Cah-CP2	HLA-DRB3*02:02	FTVNAGGQA	NGGSFTVNAGGQAGN	0.9300
Cah-CP1	HLA-DRB1*07:01	YQSVKSGGL	INRDGYQSVKSGGLA	0.9246
Cah-CP3	HLA-DRB3*01:01	LIADSGATV	GGALIADSGATVEGT	0.8974
Cah-CP4	HLA-DRB3*02:02	WIQNGGIAG	GGQWIQNGGIAGNTT	0.8965
Cah-CP3	HLA-DRB3*01:01	LIADSGATV	SGGALIADSGATVEG	0.8888
Cah-CP4	HLA-DRB3*02:02	WIQNGGIAG	TGGQWIQNGGIAGNT	0.8761
Cah-CP5	HLA-DRB1*07:01	LVTSTAATV	GGALVTSTAATVIGT	0.8712
Cah-CP6	HLA-DRB1*07:01	MTISTGLEL	ANGMTISTGLELGPD	0.8661
Cah-CP2	HLA-DRB3*02:02	FTVNAGGQA	ENGGSFTVNAGGQAG	0.8615
Cah-CP1	HLA-DRB1*07:01	YQSVKSGGL	DGYQSVKSGGLATGT	0.8483
Cah-CP5	HLA-DRB1*07:01	VTSTAATVI	GALVTSTAATVIGTN	0.8459
Cah-CP6	HLA-DRB3*02:02	LAVNTTLNN	LNGLAVNTTLNNRGE	0.8393
Cah-CP5	HLA-DRB1*07:01	LVTSTAATV	TGGALVTSTAATVIG	0.8366
Cah-CP2	HLA-DRB3*02:02	FTVNAGGQA	GSFTVNAGGQAGNTT	0.8340
Cah-CP3	HLA-DRB3*01:01	LIADSGATV	TSGGALIADSGATVE	0.8185
Cah-CP6	HLA-DRB1*07:01	MTISTGLEL	TANGMTISTGLELGP	0.8036
Cah-CP4	HLA-DRB3*02:02	WIQNGGIAG	NTGGQWIQNGGIAGN	0.8033
Cah-CP1	HLA-DRB1*07:01	YQSVKSGGL	IINRDGYQSVKSGGL	0.7937
Cah-CP7	HLA-DRB3*02:02	IAGNTTVTT	NGGIAGNTTVTTNGR	0.7793
Cah-CP3	HLA-DRB1*03:01	LIADSGATV	GGALIADSGATVEGT	0.7717
Cah-CP5	HLA-DRB3*02:02	LAVNTTLNN	SLNGLAVNTTLNNRG	0.7709

[1] A threshold value of 0.5 was used. Predicted epitopes are shown underlined in the amino acid sequence.
[2] A threshold value of 1.0 and window of 7 were used. Predicted epitopes are indicated with asterisk in the amino acid sequence.
[3] A set of seven human HLA-DR alleles were analyzed: HLA-DRB1*03:01, HLA-DRB1*07:01, HLA-DRB1*15:01, HLA-DRB3*01:01, HLA-DRB3*02:02, HLA-DRB4*01:01, HLA-DRB5*01:01. An epitope length of 15 was defined as the default parameter. Binding peptides with a score > 0.75 were selected and are shown in yellow in the amino acid sequence.

Supplementary Table 8. Prediction of potential lineal B-cell epitopes and MCH-II binding peptides in the EspA protein (Sequence ID: WP_000381516.1)

Amino acid sequence:

```
     ***********            ************************* ********
MDTSNATSVVNVSASSSTSTIYDLGNMSKDEVVKLFEELGVFQAAILMFSYMYQAQSNLSIAKFADMNEA

          **************       ***********                        ******
SKASTTAQKMANLVDAKIADVQSSTDKNAKAKLPQDVIDYINDPRNDISVTGIRDLSGDLSAGDLQTVKA

****    ********           ******
AISAKANNLTTVVNNSQLEIQQMSNTLNLLTSARSDVQSLQYRTISAISLGK
```

B-cell epitopes predicted by BepiPred 2.0[1]

Epitope ID	Sequence	Length
EspA-B1	VVNVSASSSTSTIYDLGNMSKDEVVKLFEEL	31
EspA-B2	DMNEASK	7
EspA-B3	STTAQKM	7
EspA-B4	VQSSTDKNAKAKLP	14
EspA-B5	YINDPRNDI	9
EspA-B6	TGIRDLSGDLSAGDLQ	16
EspA-B7	SAKANNLTTVVNNSQLEIQQMSN	23

B-cell epitopes predicted by Kolaskar and Tongaonker antigenicity method[2]

Epitope ID	Sequence	Length
EspA-K1	ATSVVNVSASS	11
EspA-K2	DEVVKLFEELGVFQAAILMFSYMYQ	25
EspA-K3	QSNLSIAK	8
EspA-K4	ANLVDAKIADVQSS	14
EspA-K5	AKLPQDVIDYI	11
EspA-K6	LQTVKAAISA	10
EspA-K7	LTTVVNNS	8
EspA-K8	LNLLTS	6

MHC-II binding peptides by NetMHCIIpan 4.1[3]

Core Peptide ID	Allele	Core sequence	Peptide sequence	Score
EspA-CP1	HLA-DRB1*07:01	YRTISAISL	QSLQYRTISAISLGK	0.8888
EspA-CP1	HLA-DRB1*07:01	YRTISAISL	VQSLQYRTISAISLG	0.7799

[1] A threshold value of 0.5 was used. Predicted epitopes are shown underlined in the amino acid sequence.
[2] A threshold value of 1.0 and window of 7 were used. Predicted epitopes are indicated with asterisk in the amino acid sequence.
[3] A set of seven human HLA-DR alleles were analyzed: HLA-DRB1*03:01, HLA-DRB1*07:01, HLA-DRB1*15:01, HLA-DRB3*01:01, HLA-DRB3*02:02, HLA-DRB4*01:01, HLA-DRB5*01:01. An epitope length of 15 was defined as the default parameter. Binding peptides with a score > 0.75 were selected and are shown in yellow in the amino acid sequence.

Supplementary Table 9. Prediction of potential lineal B-cell epitopes and MCH-II binding peptides in the OmpT protein (Sequence ID: WP_001201843)

Amino acid sequence:

```
    ****************
MRAKLLGIVLTTPIAISSFASTETLSFTPDNINADISLGTLSGKTKERVYLAEEGGRKVSQLDWKFNNAAI

            *******
IKGAINWDLMPQISIGAAGWTTLGSRGGNMVDQDWMDSSNPGTWTDESRHPDTQLNYANEFDLNIKGWLLN

     *******                                              *******
EPNYRLGLMAGYQESRYSFTARGGSYIYSSEEGFRDDIGSFPNGERAIGYKQRFKMPYIGLTGSYRYEDFE

                                             *********************
LGGTFKYSGWVEASDNDEHYDPGKRITYRSKVKDQNYYSVSVNAGYYVTPNAKVYVEGTWNRVTNKKGNTS

LYDHNDNTSDYSKNGAGIENYNFITTAGLKYTF
```

B-cell epitopes predicted by BepiPred 2.0[1]

Epitope ID	Sequence	Length
OmpT-B1	TETLSFTPDNI	11
OmpT-B2	LAEEGGR	7
OmpT-B3	LGSRGGNMVDQDWMDSSNPGTWTDESRH PDTQLNYA	36
OmpT-B4	YSSEEGFRDDIGSFP	15
OmpT-B5	EHYDPGKRITYRSKVKD	17
OmpT-B6	RVTNKKGNTSLYDHNDNTSDYSKN	24

B-cell epitopes predicted by Kolaskar and Tongaonker antigenicity method[2]

Epitope ID	Sequence	Length
OmpT-K1	KLLGIVLTTPIAISSF	16
OmpT-K2	MPQISIG	7
OmpT-K3	RLGLMAG	7
OmpT-K4	MPYIGLT	7
OmpT-K5	YYSVSVNAGYYVTPNAKVYVE	21

MHC-II binding peptides by NetMHCIIpan 4.1[3]

Core Peptide ID	Allele	Core sequence	Peptide sequence	Score
OmpT-CP1	HLA-DRB3*02:02	WKFNNAAII	QLDWKFNNAAIIKGA	0.9580
OmpT-CP1	HLA-DRB3*02:02	WKFNNAAII	SQLDWKFNNAAIIKG	0.9411
OmpT-CP2	HLA-DRB1*07:01	YYVTPNAKV	NAGYYVTPNAKVYVE	0.9393
OmpT-CP2	HLA-DRB1*07:01	YYVTPNAKV	VNAGYYVTPNAKVYV	0.9231
OmpT-CP3	HLA-DRB5*01:01	YLAEEGGRK	ERVYLAEEGGRKVSQ	0.9154
OmpT-CP2	HLA-DRB1*07:01	YYVTPNAKV	SVNAGYYVTPNAKVY	0.9062
OmpT-CP4	HLA-DRB5*01:01	YIGLTGSYR	KMPYIGLTGSYRYED	0.8882
OmpT-CP3	HLA-DRB5*01:01	YLAEEGGRK	KERVYLAEEGGRKVS	0.8844
OmpT-CP5	HLA-DRB1*07:01	FASTETLSF	ISSFASTETLSFTPD	0.8753
OmpT-CP1	HLA-DRB3*02:02	WKFNNAAII	VSQLDWKFNNAAIIK	0.8671
OmpT-CP1	HLA-DRB3*02:02	WKFNNAAII	LDWKFNNAAIIKGAI	0.8579
OmpT-CP2	HLA-DRB1*07:01	YYVTPNAKV	AGYYVTPNAKVYVEG	0.8430
OmpT-CP4	HLA-DRB5*01:01	YIGLTGSYR	FKMPYIGLTGSYRYE	0.8291
OmpT-CP6	HLA-DRB1*07:01	YNFITTAGL	IENYNFITTAGLKYT	0.8215
OmpT-CP2	HLA-DRB1*07:01	YYVTPNAKV	VSVNAGYYVTPNAKV	0.8089
OmpT-CP3	HLA-DRB5*01:01	YLAEEGGRK	TKERVYLAEEGGRKV	0.7926
OmpT-CP5	HLA-DRB1*07:01	FASTETLSF	AISSFASTETLSFTP	0.7857
OmpT-CP2	HLA-DRB3*02:02	YYVTPNAKV	NAGYYVTPNAKVYVE	0.7690

OmpT-CP7	HLA-DRB1*07:01	YIYSSEEGF	GGSYIYSSEEGFRDD	0.7602
OmpT-CP3	HLA-DRB5*01:01	YLAEEGGRK	RVYLAEEGGRKVSQL	0.7585

[1] A threshold value of 0.5 was used. Predicted epitopes are shown underlined in the amino acid sequence.
[2] A threshold value of 1.0 and window of 7 were used. Predicted epitopes are indicated with asterisk in the amino acid sequence.
[3] A set of seven human HLA-DR alleles were analyzed: HLA-DRB1*03:01, HLA-DRB1*07:01, HLA-DRB1*15:01, HLA-DRB3*01:01, HLA-DRB3*02:02, HLA-DRB4*01:01, HLA-DRB5*01:01. An epitope length of 15 was defined as the default parameter. Binding peptides with a score > 0.75 were selected and are shown in yellow in the amino acid sequence.

Supplementary Table 10. Lineal B-cell epitopes and MHC-II binding peptides predicted for the Chimera 3 protein.

Chimera 3			
Amino acid sequence:<br>ADCAKGKIEFSKYNENDTFTVKVAGKEYWTSRWNLQPLLQSAQLTGMTVTIKSSTCESGSGFAEVQFNNDEPDSPTTTDPDAAASATETATRDQLTKEAFQNPDNQKV<br>NIDELGNAIPSGVLKDDVVANIEEQAKAAGEEAKQQAIENNAQAQKKYDASQQTTQTIRLTVTNDCPVTITTTPPQTVGVSSTTPIGFSAKVTTSDQCIKAGAKVWLW<br>GTGPANKWVLQHAKVAKQKYTLNPSIDGGADFVNQGTDAKIYKKLTSGNKFLNASVSVNPKTQVLIPGEYTMILHAAVDFFFDELKIDNKVDIIGNNVRGELPNIWLQ<br>YGQFKLKASGGDGTYSWYSENTSIATVDASGKVTLNGKGSVVIKATSGDKQTVSYTIKAPSYMIKVDKQAYYADAMSICKNLLPSTQTVLSDIYDSWGAANKYSHYSS<br>MNSITAWIKQTSSEQRSGVSSTYNLITQNPLPGVNVNTPNVYAVCVE			
Antigenic domain	B-cell epitopes		MHC-II binding peptides
	BepiPred	Kolaskar and Tongaonker	NetMHCIIpan 4.1
Stx2B	Stx2B-B1: IEFSKYNENDTFT<br>Stx2B-B2: KEYWTSRWNLQPLLQSAQLT<br>Stx2B-B3: STCESGSGFAE	Stx2B-K1: TVKVAGK<br>Stx2B-K2: LQPLLQSAQ<br>Stx2B-K3: TIKSSTCE	Stx2B-CP1:<br>YNENDTFTV
Tir	Tir-E7: EPDSPTTTDPDAAASATETATRDQLTKEAFQN PDNQKVNIDELGNAIPSGVLKD<br>Tir-E8: KAAGEEAKQQAIENNAQAQKKYD	Tir-K11: PSGVLKDDVVANI	
AggA	AggA-B2: TTPPQTVGVSSTTPIG<br>AggA-B3: LQHAKVAKQKYTLNPSIDGGADFVNQGTDAKI YKKLTSGNKFL	AggA-K2: TNDCPVTIT<br>AggA-K3: SDQCIKAG<br>AggA-K4: KWVLQHAKVAKQK<br>AggA-K5: NASVSVN<br>AggA-K6: KTQVLIPG	AggA-CP1:<br>YTLNPSIDG<br>AggA-CP2:<br>YKKLTSGNK
Int	Int-B3: GNNVRGELPNIWLQYGQFKLKASGGDGTYSWYS ENTS<br>Int-B4: KQAYYADAMSICKNLLPSTQT<br>Int-B5: DSWGAANKYSHYSSMNSITA<br>Int-B6: QTSSEQRSGV<br>Int-B7: PLPGVNVNTPNV	Int-K5: NIWLQYGQFKLK<br>Int-K6: IATVDASGKV<br>Int-K7: KGSVVIKA<br>Int-K8: TVSYTIKAPSYMIKVDKQAYYA DAMSICKNLLPSTQTVLSDI<br>Int-K9: RSGVSSTYNLI	Int-CP1: IKVDKQAYY<br>Int-CP4: YNLITQNPL

Supplementary Table 11. Lineal B-cell epitopes and MHC-II binding peptides predicted for the Chimera 4 protein.

<table>
<tr><td colspan="4">Chimera 4</td></tr>
<tr><td colspan="4">Amino acid sequence:
TTVTTNGRQVVLEGGTASDTVIRDGGGQSLNGLAVNTTLNNRGEQWVHEGGVATGTIINRDGYQSVKSGGLATGTIINTGAEGGPDSDNSYTGQKVQGTAESTTINKN
GRQIILFSGLARDTLIYAGGDQSVHGRALNTTLNGGYQYVHRDGLALNTVINEGGWQVVKAGGAAGNTTINQNGELRVHAGGMANLVDAKIADVQSSTDKNAKAKLPQ
DVIDYINDPRNDISVTGIRDLSGDLSAGDLQTVKAAISAKKVSQLDWKFNNAAIIKGAINWDLMPQISIGAAGWTTLGSRGGNMVDQDWMDSSNPGTWTDESRHPDTQ
LNYANEFDLNIKGWLLNEPNYRLGLMAGYQESRYSFTARGGSYIYSSEEGFRDDIGSFPNGERAIGY</td></tr>
<tr><td rowspan="2">Antigenic domain</td><td colspan="2">B-cell epitopes</td><td>MHC-II binding peptides</td></tr>
<tr><td>BepiPred</td><td>Kolaskar and Tongaonker</td><td>NetMHCIIpan 4.1</td></tr>
<tr><td>Cah</td><td>Cah-B2: TNGRQVVLEGGTASDTVIRDGGGQSLNGLAVN
Cah-B3: NNRGEQWVHEGGVATGTIINRDGYQSVKSGGLA
Cah-B4: NTGAEGGPDSDNSYTGQKVQGTAESTTINKNGR QIIL
Cah-B5: AGGDQSVHGRALNTTLNGGYQYVHRDGLA
Cah-B6: NEGGWQVVKAGGAAGNTTINQNGELRVHAG</td><td>Cah-K1: RQVVLEG
Cah-K2: LNGLAVNT
Cah-K3: YQSVKSG
Cah-K4: GQKVQGT
Cah-K5: QIILFSGL
Cah-K6: RDTLIYAG
Cah-K7: DQSVHGR
Cah-K8: GYQYVHRDG
Cah-K9: WQVVKAG
Cah-K10: ELRVHAG</td><td>Cah-CP1: YQSVKSGGL
Cah-CP6: LAVNTTLNN</td></tr>
<tr><td>EspA</td><td>EspA-B4: VQSSTDKNAKAKLP
EspA-B5: YINDPRNDI
EspA-B6: TGIRDLSGDLSAGDLQ</td><td>EspA-K4: ANLVDAKIAD VQSS
EspA-K5: AKLPQDVIDYI
EspA-K6: LQTVKAAISA</td><td></td></tr>
<tr><td>OmpT</td><td>OmpT-B3: LGSRGGNMVDQDWMDSSNPGTWTDESRHPDT QLNYA
OmpT-B4: YSSEEGFRDDIGSFP</td><td>OmpT-K2: MPQISIG
OmpT-K3: RLGLMAG</td><td>OmpT-CP1: WKFNNAAII
OmpT-CP7: YIYSSEEGF</td></tr>
</table>

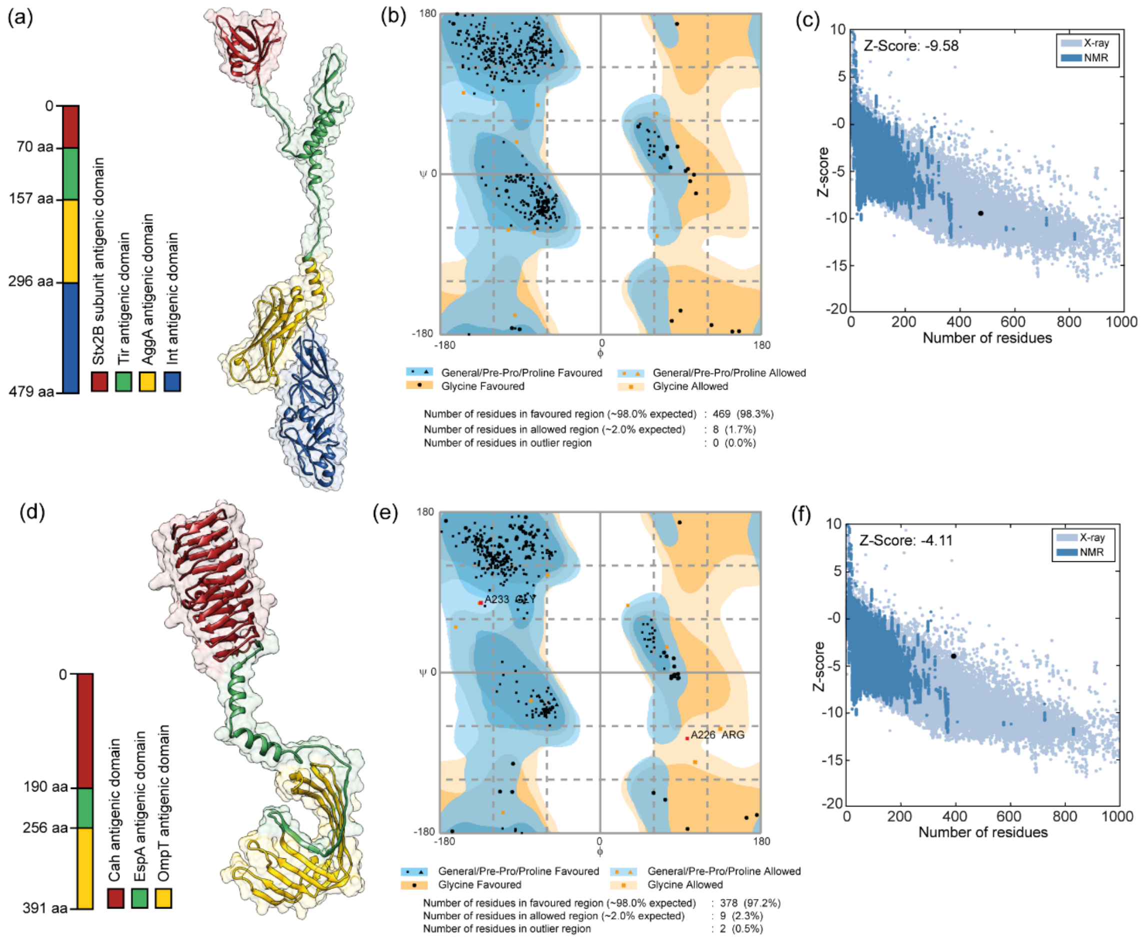

Supplementary Figure 1. *In silico* design of Chimera 3 and Chimera 4 proteins. a) Predicted 3D structure of Chimera 3 (Chi3). Antigenic domains are shown as indicated in the legend on the left. b) Ramachandran plot of Chi3 model indicating that 98.3% of amino acid residues are in favorable regions. c) Z-score plot for the Chi3 model. Dark blue and light blue regions represent Z-scores for native protein structures determined by NMR and X-ray, respectively. The black dot shows the Z-score for the Chi3 model. d) Predicted 3D structure of Chimera 4 (Chi4). Antigenic domains are shown as indicated in the legend on the left. e) Ramachandran plot of Chi4 model indicating that 97.2% of amino acid residues are in favorable regions. f) Z-score plot for the Chi4 model. The black dot shows the Z-score for the Chi4 model.
